# Rewilding soil and litter invertebrates and fungi increases decomposition rates and alters detritivore communities

**DOI:** 10.1002/ece3.11128

**Published:** 2024-03-10

**Authors:** Peter Contos, Nicholas P. Murphy, Zachary J. Kayll, Tamara Morgan, Joshua J. Vido, Orsi Decker, Heloise Gibb

**Affiliations:** ^1^ Department of Environment and Genetics, Centre for Future Landscapes, School of Agriculture, Biomedicine, and Environment La Trobe University Melbourne Victoria Australia; ^2^ Department of Microbiology, Anatomy, Physiology and Pharmacology, School of Agriculture, Biomedicine, and Environment La Trobe University Melbourne Victoria Australia; ^3^ Bavarian Forest National Park Nature Conservation and Research Grafenau Germany

**Keywords:** decomposition, ecological restoration, ecosystem processes, litter transplants, rewilding, soil inoculation

## Abstract

Habitat degradation and associated reductions in ecosystem functions can be reversed by reintroducing or ‘rewilding’ keystone species. Rewilding projects have historically targeted restoration of processes such as grazing regimes or top‐down predation effects. Few projects focus on restoring decomposition efficiency, despite the pivotal role decomposition plays in global carbon sequestration and nutrient cycling. Here, we tested whether rewilding entire communities of detritivorous invertebrates and fungi can improve litter decomposition efficiency and restore detritivore communities during ecological restoration. Rewilding was conducted by transplanting leaf litter and soil, including associated invertebrate and fungal communities from species‐rich remnant sites into species‐poor, and geographically isolated, revegetated farmland sites in a temperate woodland region of southeastern Australia. We compared communities in sites under the following treatments: remnant (conservation area and source of litter transplant), rewilded revegetation (revegetated farmland site with litter transplant) and control revegetation (revegetated site, no transplant). In one ‘before’ and three ‘after’ sampling periods, we measured litter decomposition and the abundance and diversity of detritivorous invertebrates and fungi. We quantified the effect of detritivores on the rate of litter decomposition using piecewise Structural Equation Modelling. Decomposition was significantly faster in rewilding sites than in both control and remnant areas and was largely driven by a greater abundance of invertebrate detritivores. Similarly, the abundance of invertebrate detritivores in rewilding revegetation sites exceeded the level of remnant communities, whereas there was little difference between control and remnant sites. In contrast, rewilding did not increase saprotrophic fungi relative abundance/diversity and there was no strong relationship between decomposition and fungal diversity. Our findings suggest the relatively simple act of transplanting leaf litter and soil can increase functional efficiency during restoration and alter community composition. Our methods may prove important across a range of contexts where other restoration methods have failed to restore ecosystem processes to pre‐degradation levels.

## Graphical abstract

Rewilding entire communities of invertebrates and fungi during ecological restoration increased detritivore abundances. We found this had cascading effects that enhanced the efficiency of decomposition in revegetated farmland sites.

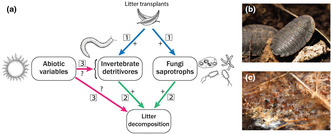

## 1 INTRODUCTION

Ecosystem processes, such as nutrient cycling and carbon sequestration, are directly threatened by land degradation (Kollmann et al., 2016). Ecological restoration aims to reverse land degradation by safeguarding and restoring processes critical to humanity. However, restoration has only recently shifted focus from reinstating historical species communities to restoring broad ecosystem processes (Wortley et al., 2013). Many restoration projects have traditionally relied on biodiversity‐ecosystem function research and assumed if biodiversity (primarily plant biodiversity) is reinstated to pre‐disturbance levels full functional recovery should follow. However, ecological restoration is rarely this straightforward as biodiversity‐ecosystem function relationships are often complex and context‐dependent (Kollmann et al., 2016). The lack of an explicit approach to restoring ecosystem function in the past may explain why restoration projects are now reporting failed functional recovery, even after relatively long periods of time (+50 years) post‐restoration (e.g. Moreno‐Mateos et al., 2012).

Litter decomposition is a critical ecosystem process yet is the focal point of only 6% of process‐driven restoration projects, with most focusing on nutrient dynamics (26%) or productivity (18%) (Kollmann et al., 2016). Up to 90% of terrestrial plant biomass escapes herbivory and enters the dead organic pool as leaf litter (Cebrian, 1999). This massive influx of nutrients must decompose before it is cycled into the soil and reabsorbed by plants. Litter decomposition is a complex process of chemical and physical breakdown driven by abiotic conditions (e.g. evapotranspiration; Berg et al., 1993), direct consumption by invertebrates and microbes (Gessner et al., 2010) and litter traits such as lignin content and nutrient concentration (Ge et al., 2013). Litter is not only a source of nutrients for plants, but provides both habitat and food for an astounding level of diversity (Fujii et al., 2020). Understanding how anthropogenic activities alter both decomposition and the diversity of organisms that drive this process is thus critical for proper ecological restoration.

Agricultural practices have lasting legacy effects that both slow decomposition and shift detritivore communities (Stone et al., 2020). For example, heavy agricultural machinery compacts soil and reduces oxygen flow between soil pores, which alters microbial activity and ultimately slows decomposition rates (Carlesso et al., 2019). Alternatively, agricultural legacies (fertiliser addition, ungulate dung) can elevate soil nutrients and increase detritivore abundance, thereby enhancing decomposition rates (Wang et al., 2019). Restoration sites are also inherently situated on former agricultural land in the most productive areas, meaning they may have the capacity for more efficient ecosystem functions as compared to target reference sites which are often less productive and were historically left uncleared (Simmonds et al., 2017). Ecological restoration can partly reverse the legacy of agriculture (Matzek et al., 2016). However, both active and passive restoration can fail to recover decomposition to pre‐disturbance levels even after multiple decades (Rowland et al., 2009; Stone et al., 2020). This presents a significant impediment to full ecosystem recovery and may necessitate a more proactive approach to recovering decomposition rates.

When restoration is exceedingly slow or fails to satisfactorily recover ecosystem processes, practitioners can turn to active reintroductions or ‘rewilding’ of functionally important fauna to address species recolonisation failures (Contos et al., 2021). Rewilding projects have historically targeted restoration of top‐down effects or grazing regimes (Pettorelli et al., 2018), with comparatively fewer projects considering decomposition (Eldridge & James, 2009). Bioturbation of the litter layer by rewilded digging mammals can increase decomposition via microclimatic alterations (Decker et al., 2019). Alternatively, rewilding can slow decomposition if the reintroduced species exerts top‐down effects on primary detritivores such as termites (Coggan et al., 2016). There is a significant knowledge gap surrounding our ability to restore decomposition through rewilding. Addressing this will involve exploring other elements of the community with the potential to alter decomposition.

The selection of a species community to attain target functional recovery is not trivial as multiple species may drive a specific process (Carlucci et al., 2020). However, we know that invertebrates and microbes that live within the litter layer are key drivers of decomposition. Invertebrates are responsible for up to ~31% of decomposition in forests where they directly consume and ‘shred’ litter (Xu et al., 2020). Similarly, microbes are fundamental in the initial conditioning of litter and expression of extracellular enzymes that break down plant tissue (Krishna & Mohan, 2017). Despite their abundant contributions to decomposition, invertebrate and microbial communities have never been actively reintroduced to enhance decomposition rates (Contos et al., 2021).

Invertebrate and microbial rewilding is an emerging field yet has already shown numerous practical applications in restoring ecosystem function (Contos et al., 2021). Actively reintroducing invertebrates and microbes during restoration can increase both soil stability (Faist et al., 2020) and alter the succession and trajectory of plant communities (Wubs et al., 2016). Invertebrates and microbes are prime candidates for rewilding projects as they are often dispersal‐constrained and struggle to reach and exert their ecosystem services on newly restored habitat without assistance (Contos et al., 2024). However, their efficacy in restoring decomposition is currently unclear.

Choosing a subset of invertebrates and microbes that will enhance decomposition rates is an incredibly complex task as they have many inter‐dependencies. Litter‐dwelling invertebrates and microbes both work in tandem to break down litter (e.g. microbial conditioning of litter which increases palatability for invertebrates) and exhibit intra‐community predation (e.g. invertebrates grazing on microbes) (Krishna & Mohan, 2017). Despite extensive research, there is still no general pattern for which groups are more likely to drive decomposition (Hättenschwiler et al., 2005). Functional redundancy is also rife within invertebrates and microbes with decomposition often supported by a suite of species (Ferrer et al., 2020). Reintroducing the entire community may therefore be more appropriate; a practice that is unique to invertebrate and microbial rewilding projects. Termed ‘whole‐of‐community’ rewilding (Contos et al., 2021), this practice allows practitioners to reintroduce entire communities (usually contained in subsets of habitat such as soil or litter) rather than rely on species‐by‐species reintroductions common to traditional (vertebrate) rewilding projects (Contos et al., 2023). Despite their potentially useful application, only two studies have attempted to reintroduce whole litter communities with neither linking to decomposition (Benetková et al., 2020; Contos et al., 2023).

**FIGURE 1:**
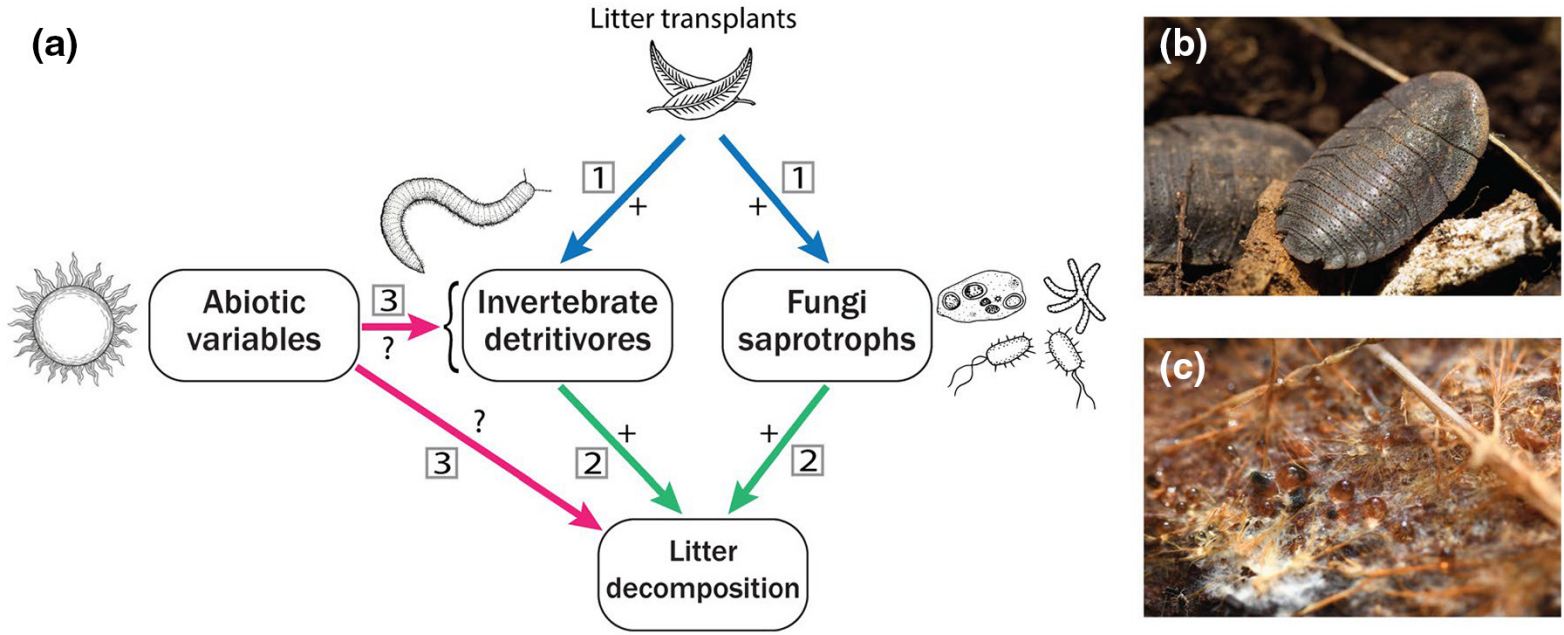
Hypotheses of the predicted effect of litter transplants on detritivore communities and litter decomposition (a). Hypothesis 1 (blue arrows) predicts that rewilding via litter transplants would increase invertebrate and fungal saprotroph abundance and diversity. This will then have a cascading effect (hypothesis 2, green arrows) whereby increased abundances/diversity of detritivores results in faster litter decomposition rates. Hypothesis 3 (pink arrows) predicts possible direct and indirect effects of environmental variables on decomposition and were expected to vary seasonally. Invertebrates and fungi are key decomposers ‐ such as *Laxta* cockroaches (b) (Photo credit: L Menz) and fungal species that colonise wood and litter (c).

Here we test whether leaf litter transplants are effective in reintroducing detritivore communities and enhancing decomposition rates during ecological restoration. We moved leaf litter (with entire communities in situ) from species‐rich remnant sites, into species‐poor and geographically isolated revegetated sections of farmland. This study is part of a long‐term experiment, and we have previously reported increases in beetle species richness post‐rewilding (Contos et al., 2023). We measured pre‐ and post‐rewilding detritivore communities in conjunction with decomposition rates in remnant sites, inoculated revegetation sites and control revegetation sites. We hypothesised that rewilding via litter transplants would increase litter decomposition in the transplant sites, relative to controls, by increasing the diversity and abundance of detritivorous invertebrates and saprotrophic fungi (Figure 1). If decomposition in control sites was less than that in remnant sites, we would expect that transplants would raise the level to that of remnant sites; if decomposition in control and remnant sites was similar, we would expect that decomposition rates in transplant sites would exceed those of remnants (by increasing complementarity of detritivores and saprotrophic fungi).

## 2 MATERIALS AND METHODS

### 2.1 Study site and experimental design

We situated our study in an agricultural area in southeastern Australia (37°0′20.76″ S, 145°28′6.05″ E), where sections of pasture had been revegetated using indigenous tree species. The remaining unaltered remnant areas were typified by *Eucalyptus* woodlands. The study area has a Mediterranean climate with dry, hot summers and wet, cool winters.

**FIGURE 2:**
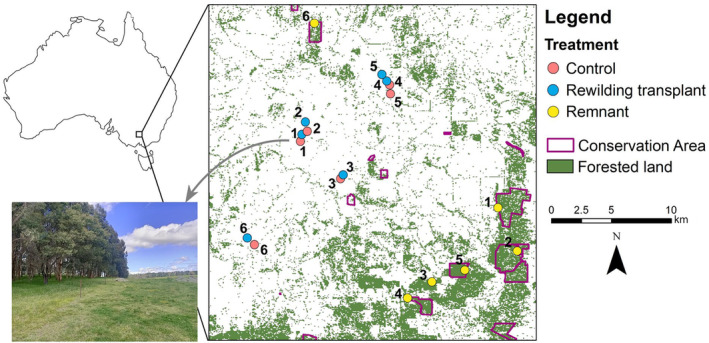
Distribution of treatments and sites (with their replicate number) within the study. A typical revegetation site is shown in the bottom left.

We sampled 18 sites within this region with 6 blocked replicates of three treatments: ‘remnant’ (uncleared conservation area), ‘control’ (revegetated pasture with no transplant) and ‘rewilding transplant’ (revegetated pasture with a litter transplant). Due to historical biases in clearing patterns within Australia (Simmonds et al., 2017), remnant sites tended to be higher in elevation and steeper in aspect as compared to revegetation sites (Table S1). Within each block, a paired remnant site was the source of the transplant and was grouped with spatially paired revegetation sites. However, remnant sites were spatially separated from revegetation pairs and geographically clustered (within ~20 km of one another) (Figure 2). Revegetated sites were historically cleared for agriculture and then replanted using native tree and shrub tubestock 14–22 years prior to the commencement of this study. Sites within this age range had a developed leaf litter layer, providing critical habitat for transplanted litter‐dwelling detritivores. We constructed a 10 × 10 m quadrat within each of the 18 sites, around which we centred our sampling. Within remnant sites, the quadrat was placed 20–50 m from the roadside and within revegetation sites, the quadrat was placed as close to the centre of the site as possible.

### 2.2 Rewilding using litter transplants

Litter transplants contained leaf litter from remnant sites as these are considered ‘target’ states, that is, sites containing species that may have existed in the restoration area pre‐degradation (Mcdonald et al., 2016). This allowed us to both steer the similarity of the rewilding community towards the remnant community and ensured that we transplanted species with appropriate functional traits (Jackrel et al., 2019). We used data from a previous study (Grubb et al., unpublished data) to determine that the viability of transplanted populations was likely if each transplant event consisted of 10 litter samples, each measuring 80 cm litter × 80 cm litter × 5 mm soil depth (0.0032 m^3^). We included the thin layer of topsoil at the interface of the litter layer as litter‐dwelling species often reside at this soil depth during dry conditions.

To account for the experimental addition of leaf litter we took baseline surveys of litter depth and cover at each site to ascertain the volume of habitat added to the rewilding transplant sites during each event. Each transplant event introduced approximately 0.052 m^3^ of litter into the experimental grid which contained approximately 0.476 m^3^ of pre‐existing litter, resulting in an ~11% increase in habitat volume. Although past studies have shown litter transplants increase humidity and attract highly mobile species such as carabid beetles from surrounding areas, our 11% increase in habitat is markedly less than past studies that intentionally altered microhabitat conditions via large additions of litter (Koivula et al., 1999; Magura et al., 2005) and likely did not attract additional species to the experimental grid (Contos et al., 2023). Litter and soil samples for monitoring were taken outside the area treated with litter, further ensuring potential microclimatic changes and the minor increase in litter volume inside the experimental grid did not bias the communities sampled.

We conducted the first leaf litter transplant in November 2018 and repeated transplants in June 2019 and May 2020 (Figure S1). Repeat events over different seasons increased the likelihood of capturing all community components as leaf litter invertebrates and microbes have high spatiotemporal turnover (Beng et al., 2016; Buscardo et al., 2018). Litter samples were collected and transplanted on the same day and were kept cool during transport to reduce mortality, which was likely <5% for invertebrates (Contos et al., 2023). We spaced the 10 litter samples 1 m apart from each other on top of the in situ litter layer within the experimental grid at rewilding transplant sites. During each subsequent transplant event, we ensured new litter samples were not placed onto a previous transplant sample, thus ensuring an even distribution of transplanted litter.

### 2.3 Invertebrate surveys

We sampled communities once pre‐treatment (November 2018), 3 months post‐rewilding (January 2019), 19 months post‐rewilding (May 2020) and 35 months post‐rewilding (September 2021). Because litter was transplanted multiple times at each treatment site, we were unable to separate the effects of rewilding from that of the number of transplant events. However, the most effective methodologies for litter transplants have not yet been tested, so the focus of this study was on maximising transplant success (Contos et al., 2023). We focussed our sampling effort on macroinvertebrates (>5 mm in size) as the role mesoinvertebrates (<5 mm in size: mites, springtails etc.) play in decomposition is contentious (Kampichler & Bruckner, 2009) and their abundance and diversity make physical sorting time and cost‐intensive. We were, however, able to utilise data from a previous experiment (Contos et al., 2024) that contained mesoinvertebrate detritivore communities (Collembola and Oribatida) 19 months post‐rewilding, and these were included in the relevant statistical analyses described below. We sampled macroinvertebrate detritivore communities by scraping 25 cm × 25 cm leaf litter samples with 5 mm of the underlying soil into zip‐locked bags. Eight of these sub‐samples were taken 2 m from the experimental grid at each of the 18 sites. We sampled communities 2 m from the grid to not only avoid sampling from the slightly larger litter loads within the grid (11% increases), but to test whether invertebrates were dispersing within their new habitat. Sampling for the May 2020 period occurred before the transplant event described previously.

We used Tullgren funnels with 25 W lamps to extract live animals from leaf litter into 100% ethanol. Each sub‐sample was placed into a single Tullgren funnel and left for 7 days to ensure that the leaf litter dried completely, and all animals were expelled into the ethanol. We sorted invertebrates into morphospecies and the finest taxonomic resolution, given the available keys (mostly Family/Genus level). We assigned morphospecies to a trophic group (detritivore or other, e.g., predator, herbivore, omnivore, parasitic, scavenger) based on external morphology and available literature (Table S4). Once sorted, morphospecies counts from each sub‐sample were combined at each site to create one community sample, totalling 18 community samples per sampling period. From each community sample, we calculated the abundance and number of species (species richness) of invertebrate detritivores.

### 2.4 Fungal surveys, sequencing and bioinformatics

We measured soil fungal saprotroph communities in conjunction with macroinvertebrates. We excluded bacterial communities from the analyses due to the difficulty in assigning trophic roles to bacteria, although we do note that bacteria can be key players in decomposition (Jackrel et al., 2019). We sampled communities by removing the leaf litter layer and taking a ~50 mL soil sample 5 cm deep 2 m from each corner of the experimental grid at each site. Each sub‐sample was then combined and mixed in a plastic bag. Samples were stored in cool storage in the field and then moved into −20°C freezer before processing. We extracted community genetics using a DNeasy Powersoil Pro Kit (Qiagen) according to the manufacturer's instructions. Fungal genes were sequenced with 10% phiX control on the Illumina MiSeq platform (2 × 300) to determine diversity following an already established protocol (Illumina, 2013). The ITS2 region was targeted with primers FITS7 (GTGARTCATCGAATCTTTG)/ITS4 (TCCTCCGCTTATTGATATGC) (Ihrmark et al., 2012; Lear et al., 2018).

Bioinformatics on fungal community sequences were performed via QIIME2 2020.2 (Bolyen et al., 2019). Single‐end sequences were demultiplexed prior to denoising, truncation and chimera removal using DADA2 (via q2‐dada2) (Callahan et al., 2016). A total of 1187 unique fungal amplicon sequence variants (ASVs) were identified. Taxonomy was assigned to fungal sequences using the q2‐feature‐classifier (Bokulich et al., 2018) classify‐sklearn taxonomy classifier against the UNITE v.8.0 Dynamic classifier (i.e. either a 97% or 99% threshold based on which is more accurate for certain lineages of fungi, determined manually by experts in the field) (Nilsson et al., 2019).

Utilising the fungal genus classification, FungalTraits was used to assign ecological lifestyle information using the methodology outlined in Põlme et al. (2020). This resulted in a functional abundance table where the genus classification was replaced with ecological information. Data was then filtered to exclude global singletons (removing 224 ASVs from the original 1187 ASVs), then standardised with total sum scaling using the ‘transform_sample_counts’ function in the R‐package ‘phyloseq’ (McMurdie & Holmes, 2013) to produce relative abundances and Shannon's Diversity index using the ‘estimate_richness’ function. We filtered out any fungi which were not classified as saprotrophic, resulting in a functional abundance table comprised only of the saprotrophic community (324 ASVs).

### 2.5 Leaf decomposition monitoring

In parallel with detritivore community monitoring, we measured leaf litter decomposition using leaf litter bags that contained litter from *Eucalyptus* species in remnant sites. Decomposition may be influenced by litter lignin content and the diversity of canopy litter sources (Ge et al., 2013). These were not measured in our analyses as it was outside the scope of the study however canopy tree species were similar across remnant sites (Table S1). We only included dry, brown leaf litter and ignored green leaves and leaves with evidence of damage, herbivory, galls and late‐stage decomposition. We collected leaves from each remnant site separately such that each revegetation site received leaf litter from its paired remnant site, that is, remnant site 1 leaf litter was used for control site 1 and rewilding transplant site 1 and so forth. Once collected, we sterilised the litter using an Atherton autoclave set to dry run porous cycle at 121°C for 20 min and then oven‐dried the litter at 70°C for 7 days.

We constructed leaf litter bags measuring 18 cm × 16 cm using plastic mesh (8 mm) that allowed macroinvertebrates access. We added 8 g (±1 g) of dried litter to each bag and recorded the total weight. After the initial rewilding event, we measured decomposition on three separate occasions: summer (November 2018–January 2019), winter (May–July 2020) and spring (September–November 2021). On each occasion, we placed 8–10 litter bags 2 m and 10 m from the experimental grid. The first measurement of decomposition included 10 bags per cluster (360 in total), but power analysis indicated only 8 bags were necessary, so we used 8 bags per cluster (288 in total) in the 2020 and 2021 sampling sessions. Sampling across different seasons was necessitated by severe COVID‐19 lockdown restrictions in Melbourne in 2020 and 2021, but also enabled us to capture possible fluctuations in decomposition efficiency across treatments and seasons.

Leaf litter decomposition can be influenced by climatic and habitat features (e.g. rainfall, canopy cover) (Krishna & Mohan, 2017). We therefore measured environmental variables parallel to decomposition. During decomposition monitoring, we took long‐term measurements of temperature and humidity using deployed Tiny Tags (Gemini Data Loggers) placed in the centre of the experimental grid at each site. We also recorded average litter cover (%) at each site within a 4 m × 4 m quadrant every 10 m along a 50 m transect.

We pinned litter bags on top of existing soil and leaf litter to ensure full contact with the substrate. We collected the bags after 3 months in the field and oven dried them at 70°C for 7 days. Percentage mass loss was calculated using before and after measurements, which were then averaged across all bags in the 2 m or 10 m cluster at each site. We excluded bags from analysis that were found unpinned from the substrate or damaged. ‘Decomposition’ was defined here as the removal of leaf litter mass from the soil surface via invertebrate and microbial consumption, and did not include the mineralisation of invertebrate detritivore faeces and assimilation into the soil as this was outside the scope of this study (David, 2014).

### 2.6 Data handling and statistics

All statistical analyses were performed within the R environment (R Core Team, 2021). To test how decomposition varied across treatment, sampling period and distance (2 or 10 m from the grid), we constructed a linear mixed model in the ‘lme4’ package (Bates et al., 2015). We used ‘percentage mass loss’ as the response variable with the main effects of ‘treatment’, ‘sampling period’, ‘distance’, an interaction between these variables and ‘site’ as a random factor. Where main effects were significant, we used a post hoc estimated marginal means test in the ‘emmeans’ package (Lenth, 2021) to provide greater insight into differences between levels within a main effect.

We assessed the direct and indirect effects of environmental variables and detritivore communities on leaf litter decomposition by constructing piecewise Structural Equation Models (SEM) in the ‘piecewiseSEM’ package (Lefcheck, 2016). Each sampling session was analysed independently with composite models consisting of three equations (Figure S2). We included temperature, humidity and litter cover as environmental covariates. We used invertebrate abundance with saprotrophic fungi Shannon's diversity as opposed to invertebrate richness with saprotrophic fungi relative abundance as the inclusion of the former improved model fit. Further, invertebrate abundance is a more direct link to the consumption of litter as opposed to invertebrate richness. Similarly, the use of fungi diversity as opposed to relative abundance is a more meaningful ecological measurement as diversity measurements are less prone to the biases associated with metagenomic datasets (Bardenhorst et al., 2022). We set ‘treatment’ as an ordinal variable along the predicted restoration trajectory (control = 1, rewilding transplant = 2, remnant = 3). We ensured multi‐collinearity did not adversely affect the model by calculating the Variance Inflation Factor in each model pathway and removing each variable that was >3 (Zuur et al., 2010). We assessed the fit of each piecewise SEM using Fisher's *C* statistic and reported standardised effect sizes for variables with significant or marginally significant relationships. For the 19 months post‐rewilding piecewise SEM we included mesoinvertebrate detritivore abundance (as this data were available from a previous study, see Contos et al., 2024) and tested whether their inclusion affected decomposition.

We tested whether the abundance and species richness of macroinvertebrate detritivores responded to the treatments using generalised linear mixed models (GLMMs) in the ‘lme4’ package (Bates et al., 2015). We fitted negative binomial models on untransformed data as this is appropriate for discrete ecological count or abundance measures (O'Hara & Kotze, 2010; Stoklosa et al., 2022). We used fixed effects of ‘treatment’, ‘sampling period’ and the interaction between these two to observe how macroinvertebrate species richness and abundance changed over time and included ‘site’ as a random effect. We ran two models, one with each time point and one that excluded pre‐rewilding communities to observe the combined effects over time post‐treatment. We analysed saprotrophic fungal data in a similar manner but instead used the response variables of ‘relative abundance’ and ‘Shannon's diversity’. We used Shannon's diversity for saprotrophic fungi as opposed to richness as richness is more prone to the biases associated with metagenomic datasets, such as pipeline bioinformatic choices and amplification success (Bardenhorst et al., 2022). Fungal data models were fitted with Gaussian ‘log’ (link) functions as the dependent variables were non‐discrete.

In addition to our broad taxon analyses, we analysed finer‐scale changes of macroinvertebrate morphospecies and saprotrophic fungal ASVs across treatments using a Hierarchical Modelling of Species Communities (HMSC) approach. HMSC is a joint species distribution model (JSDM) which includes a hierarchical layer asking how species responses to environmental covariates or treatments depend on species traits and phylogenetic relationships (Tikhonov et al., 2020). Our models included the predictors ‘treatment’, environmental covariates of ‘temperature’ and ‘humidity’ (both fixed) and ‘site’ (random). We ran separate models for both macroinvertebrates and saprotrophic fungal ASVs and each sampling session to avoid model overfitting. Environmental covariates were included to determine if species distributions were better explained by environmental factors as opposed to the experimental treatment. We constructed phylogenetic relationships based on the finest taxonomic resolution of each morphospecies or ASV using the package ‘rotl’ (Michonneau et al., 2016). We ran abundance conditional‐on‐presence models and used log‐transformed abundance for macroinvertebrates, and relative abundance for fungal ASVs. We excluded rare morphospecies (present at <2 sites in each respective season) as within JSDMs rare species contribute little to model accuracy (Clark et al., 2017). We fitted HMSC models with the R‐package ‘Hmsc’ (Tikhonov et al., 2020). We sampled the posterior distribution with four Markov Chain Monte Carlo (MCMC) chains, each of which was run for 5000 iterations, of which the first 2500 were removed as burn‐in. The chains were thinned by 10 to yield 250 posterior samples per chain. We then counted the proportion of species that showed a positive or negative response to each treatment pairing with at least 90% posterior probability.

## 3 RESULTS

### 3.1 Decomposition efficiency

**FIGURE 3:**
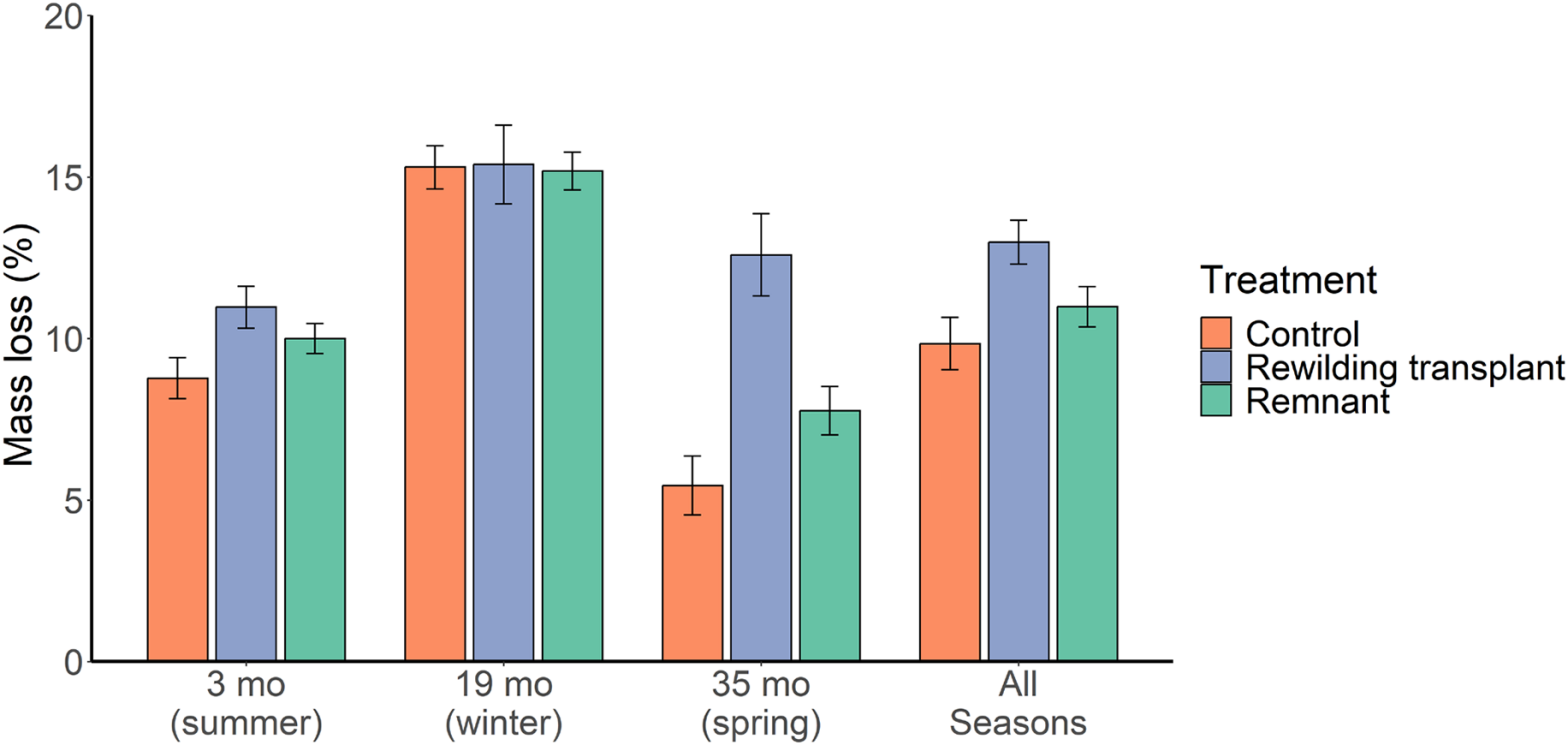
Mass lost (%) (± SE) from leaf litter bags combined at both near (2 m) and far (10 m) from the experimental grid (total bags: summer: *n* = 327, winter: *n* = 253, spring: *n* = 268).

The speed of decomposition varied across both treatment (*χ* ^2^ = 26.14, *p* < .001) and time (*χ* ^2^ = 129.87, *p* < .001) but not distance (*χ* ^2^ = 0.46, *p* = .50). Across all seasons, decomposition was significantly faster in the rewilding transplant as compared to both the control and remnant sites, whereas there was no overall difference in the control‐remnant comparison (Figure 3, Table S2). There was a significant interaction between treatment and time (*χ* ^2^ = 23.80, *p* < .001). Rewilding transplant sites tended to have faster rates of decomposition as compared to control sites 3 months post‐rewilding, whereas there were no differences among treatments 19 months post‐rewilding (Table S3). However, at 35 months post‐rewilding we found strong support for an increase in decomposition in the rewilding transplant sites as compared to both the control and remnant sites. Within this season decomposition was marginally slower in the control than the remnant.

### 3.2 Community responses

**FIGURE 4:**
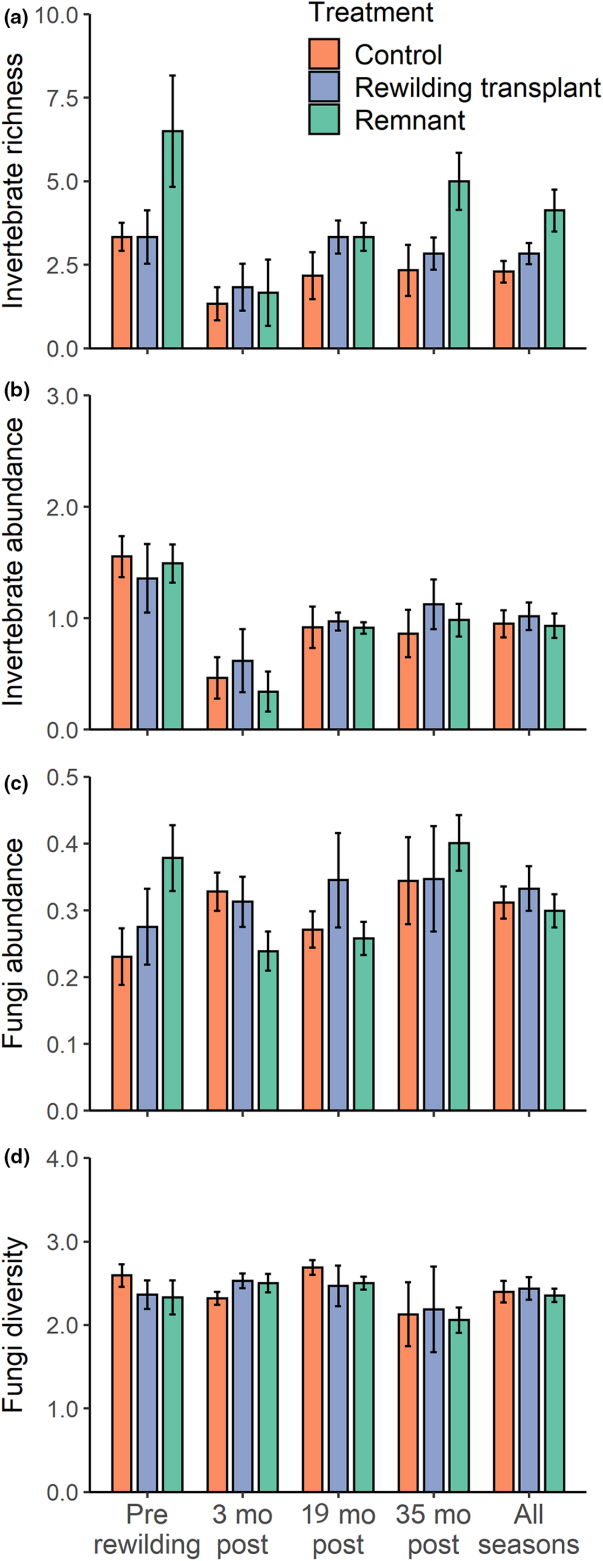
Mean invertebrate detritivore species richness (a), abundance (b), fungal saprotroph relative abundance (c) and Shannon's diversity (d) (±SE). Macroinvertebrate abundance was log + 1 transformed for graphical representation.

We collected 1707 individual invertebrate detritivores from 36 morphospecies (Table S4). The most abundant groups were Diplopoda (75.3%), Coleoptera (8.6%) and Blattodea (6.3%). Analyses of macroinvertebrate morphospecies richness revealed a main effect of ‘treatment’ in the combined post‐rewilding GLMM (*χ* ^2^ = 6.61, *p* = .04). Post hoc analyses showed species richness was indistinguishable between rewilding transplant and control sites (Figure 4a, Table S5). Control sites had marginally fewer detritivore species than remnant sites, whereas species richness was indistinguishable between the rewilding transplant and remnant sites. This suggests rewilding transplant sites had an intermediate number of detritivore species in comparison to the control and remnant.

Invertebrate detritivore abundance varied over time in the combined post‐rewilding model (*χ* ^2^ = 7.86, *p* = .02) with evidence for a marginally non‐significant difference among treatments (*χ* ^2^ = 5.65, *p* = .06). Post hoc analyses showed rewilding transplant sites had marginally greater abundances of macroinvertebrate detritivores than remnant sites (Figure 4b, Table S5). While transplants also tended to support more macroinvertebrate detritivores than controls, this difference was not significant.

Fungal saprotroph relative abundance did not vary across treatment (*χ* ^2^ = 2.01, *p* = .37) but did vary significantly over time (*χ* ^2^ = 8.08, *p* = .02) in the combined post‐rewilding model (Figure 4c). Similarly, there was no significant treatment effect on fungal saprotroph diversity in the combined post‐rewilding model (*χ* ^2^ = 0.09, *p* = .95), but a significant effect of time (*χ* ^2^ = 9.51, *p* = .009).

### 3.3 Morphospecies responses

**FIGURE 5:**
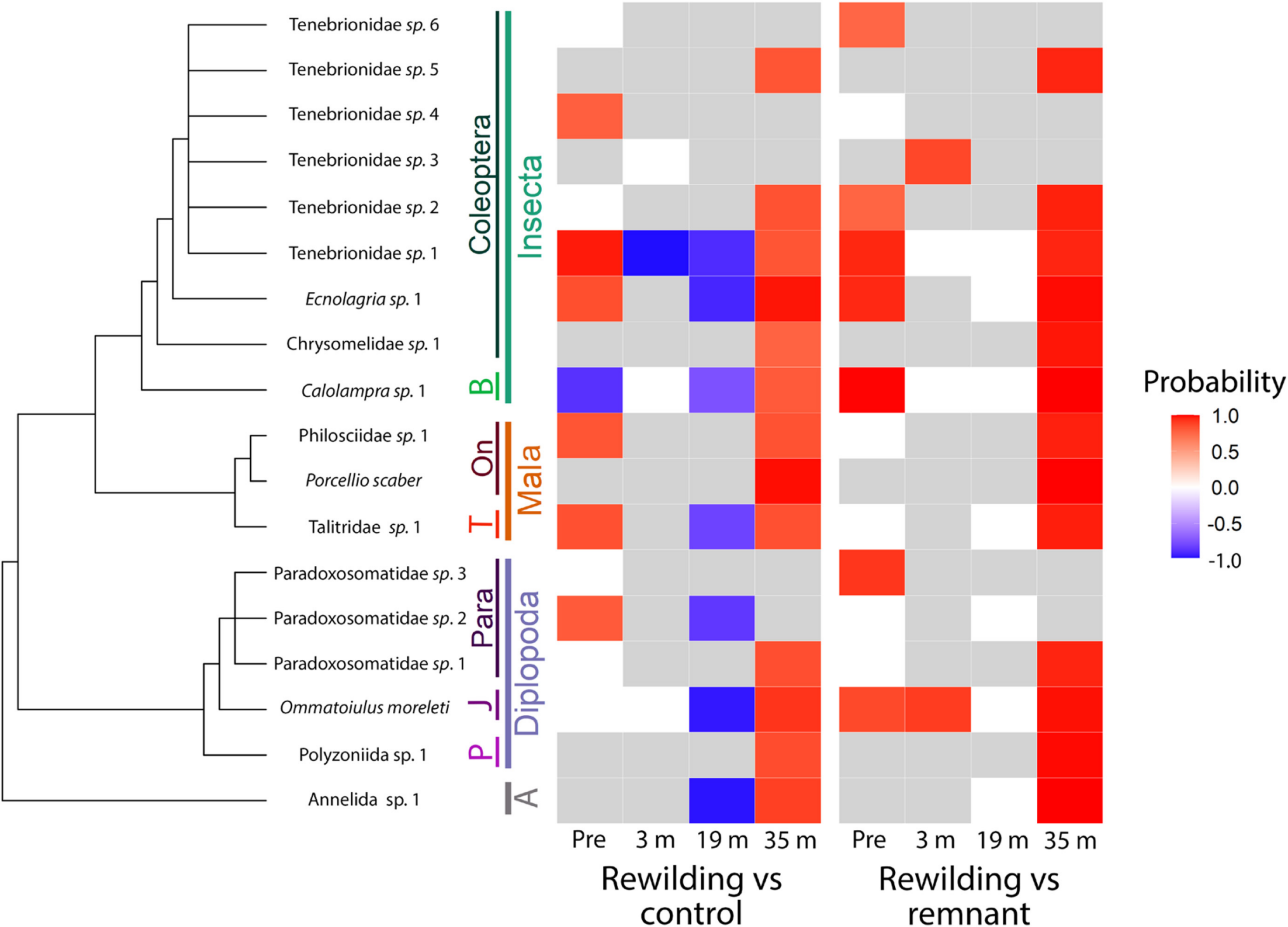
Morphospecies/species responses to the treatment pairings in the HMSC models across each sampling time point. Blue colours indicate negative responses (i.e. significantly less abundant in rewilding transplant vs other treatments), red colours indicate positive responses (i.e. significantly more abundant in rewilding transplant vs other treatments) with ≥0.75 posterior probability, white colours show species that did not gain strong statistical support and grey colours indicate a missing species in the sampling session. We relaxed the posterior probability to 75% within this graph to show overall trends in the data. A phylogenetic tree shows taxonomic relationships between species. Shortened labels: A = Annelida; B, Blattodea; J, Julidae; Mala, Malacostraca; On, Oniscidea; P, Polyzoniida; Para, Paradoxosomatidae; T, Talitridae.

The proportion of variance explained by the effect of ‘treatment’ increased over time in the macroinvertebrate HMSC models (Table S6). This suggests an increasing importance of the experimental treatment in shaping species distributions as opposed to environmental variables. The percentage of species that were significantly different in the rewilding transplant and control comparison increased over time. In the pre‐rewilding model only 8% of morphospecies were significantly different between these two treatments (>90% posterior probability), with this increasing to 25%, 57% and 23%, in the 3‐, 19‐ and 35‐month post‐rewilding models (Figure 5, Table S7). In the rewilding transplant–remnant comparison, the percentage of species that were significantly different with >90% posterior probability decreased over time before a sharp increase in species responses in the 35‐month post‐rewilding model. These species responses were all positive, that is, the species were more abundant in the rewilding transplant relative to the remnant. The same strong positive response in species abundances can be seen in the rewilding transplant relative to control at 35 months post‐rewilding.

There were few clear patterns in ASV relative abundance of fungal saprotrophs in the HMSC models. ASVs rarely expressed the same response to treatments over time and were often only found in a single sampling session (Figure S3).

### 3.4 Structural equation models

**FIGURE 6:**
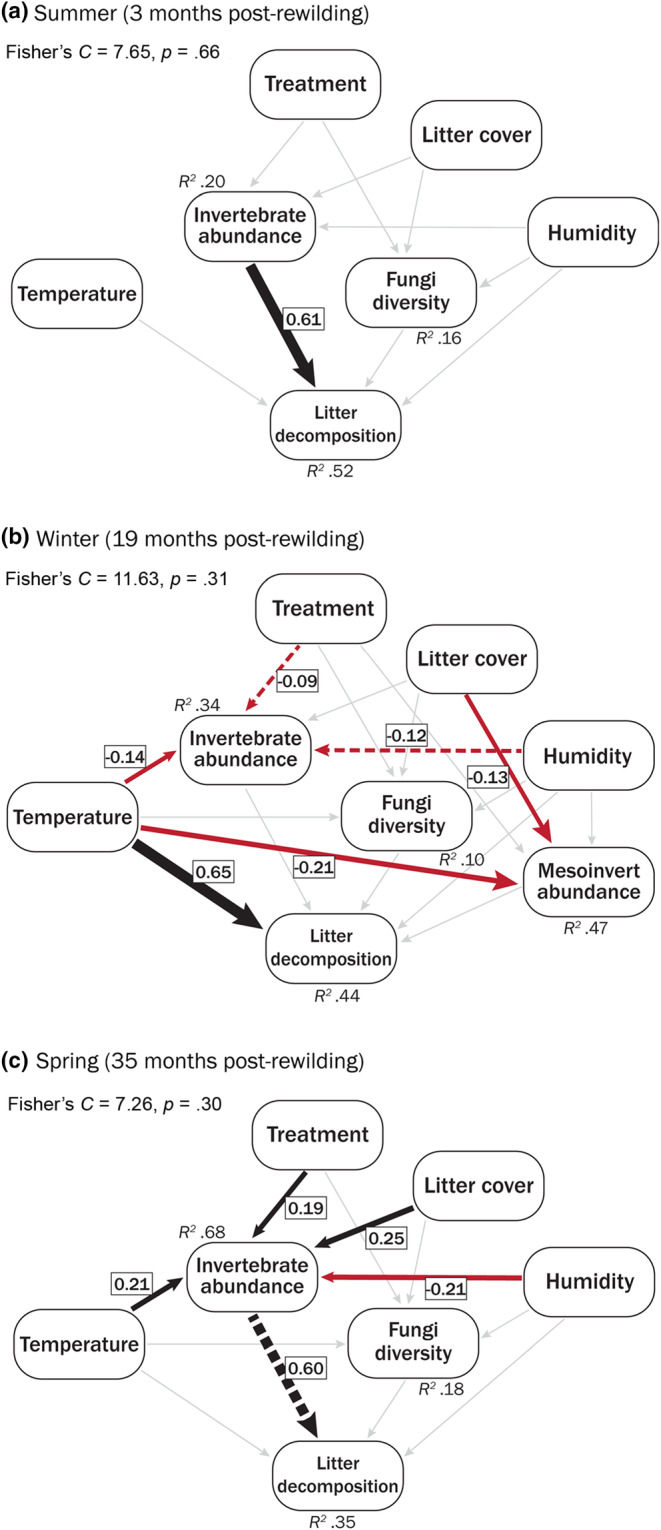
Piecewise SEMs showing relationships among measured variables across the three sampling points at: (a) 3; (b) 19 and (c) 35 months post‐rewilding. Arrows indicate the direction of the relationship, where black and red lines are positive and negative relationships, respectively. Solid lines are significant relationships, dashed lines are marginally significant trends (.05 < *p* < .1) and grey lines are non‐significant relationships that were included in the model. The thickness of the arrows indicates the magnitude of the standardised regression coefficient, which is included in the box for significant and marginally significant relationships.

The piecewise SEMs differed among the three sampling periods, with the 3‐month and 35‐month periods showing more similar results. At 3 months post‐rewilding, the model explained 52% of the variation in litter decomposition rates (Figure 6a) and the abundance of macroinvertebrate detritivores had a positive effect on leaf litter decomposition. At 35 months post‐rewilding, the model explained 35% of the variation in litter decomposition (Figure 6c). As in the 3 months post‐rewilding model, we found evidence that litter decomposition was positively associated with the abundance of macroinvertebrate detritivores. However, invertebrate abundance was also influenced by all abiotic variables measured. At 19 months post‐rewilding, the model explained 44% of the variation in litter decomposition (Figure 6b). However, decomposition was solely driven by site temperature in a positive direct relationship. Both the abundance of mesoinvertebrates and saprotrophic fungi diversity tended to increase decomposition rates although these patterns were not significant (Table S10).

## 4 DISCUSSION

Restoring ecosystem processes to pre‐disturbance levels is a challenging yet fundamental practice. Our active restoration approach using soil and leaf litter transplants altered both ecosystem function and the detritivore community during ecological restoration. This is in agreement with past studies that show transplanting entire habitats can alter invertebrate community composition and increase the efficiency of ecosystem processes (Contos et al., 2023; Wubs et al., 2016). We found support for our hypotheses as both decomposition and invertebrate abundance increased due to litter transplants, however, they increased beyond the level found in target remnant sites. Piecewise SEMs showed that decomposition was driven by macroinvertebrates in warmer months (and indirectly by abiotic variables) and solely by temperature during winter. Overall, our findings show that whole‐of‐community rewilding can be applied as a tool to steer functional community composition during restoration and restore or even enhance ecosystem processes.

### 4.1 Litter transplants improve functional efficiency

Our results showed litter decomposition speed increased in response to litter transplants. This varied seasonally (although seasonal interpretations were confounded by multiple transplant events), but overall decomposition was faster in rewilding transplant areas as compared to both control and remnant sites. This supports our initial hypothesis as decomposition exceeded the level found in remnant areas and there were few differences in decomposition between control revegetation and remnant sites. Restoration sites often have lower decomposition speeds as compared to target remnant areas (Munro et al., 2012). However, restoration sites were situated in inherently more productive and nutrient‐rich land than remnant areas which are often left uncleared as they are less productive (Simmonds et al., 2017). This suggests that agricultural restoration sites have the capacity for higher rates of decomposition than remnant sites because nutrient‐rich sites are targeted for agriculture and remaining uncleared land is often low in nutrients (Ge et al., 2013). Agricultural legacies and leaking of fertilisers from nearby pastures can further increase the potential for decomposition rates to exceed pre‐degradation levels. For example, pasture amendments via fertiliser addition result in elevated soil Phosphorus levels that last more than a decade post‐revegetation (Parkhurst et al., 2022), can increase invertebrate detritivore abundance (Clay et al., 2014) and enhance decomposition rates (Ge et al., 2013), depending on baseline soil chemistry.

Higher productivity and legacy effects of restoration sites may increase the potential for higher decomposition rates than target remnant sites, but this may not be achievable due to depauperate biota. Many invertebrates are wingless and poor dispersers, so may struggle to recolonise isolated restoration sites surrounded by cleared grazing pastures, leading to lower‐than‐expected diversity (Contos et al., 2023). Microbes also differ in their dispersal capacity according to the presence or absence of traits suited for dispersal, such as pigment production and spore formation (Choudoir et al., 2018). Within invertebrates, detritivores such as millipedes are often slow to recolonise early‐stage restoration sites compared with more mobile groups like winged predatory carabid beetles (Magura et al., 2015). We found reduced invertebrate detritivore species richness in control revegetation relative to remnant sites, although abundances were comparable. The addition of whole communities of invertebrates likely reintroduced some less dispersive remnant species and this is reflected in comparable levels of species richness in remnant and rewilding transplant sites. By adding new remnant species on top of in situ revegetation species, we may have introduced new species with complementary functional traits that allowed the detritivore community to better exploit the potentially elevated nutrients present in ex‐agricultural areas, leading to elevated decomposition rates (Cadotte et al., 2011).

### 4.2 Which groups drove decomposition?

Our results reveal two important findings regarding both the efficacy of litter transplants and the pathways that drove decomposition. First, litter transplants had little effect on saprotroph fungal communities, but a pronounced effect on macroinvertebrate detritivores at both the community and species level. This contrasts previous studies that show entire habitat additions of litter or soil can increase microbial activity during restoration in both established plots (Benetková et al., 2020) and plots where soil communities were removed prior to transplant (van der Bij et al., 2018). Our narrow focus on saprotrophs may have missed changes in the broader fungal community and is an avenue for future exploration. Second, decomposition was mainly driven by invertebrate abundance (although there was a marginal effect from fungal diversity 19 months post‐rewilding). This conclusion is supported both directly in the piecewise SEMs but also indirectly from the HMSC models, where the strongest positive macroinvertebrate responses in the rewilding transplant align with the fastest decomposition rates 35 months post‐rewilding. Our conclusion that invertebrates drive decomposition in this system is further supported by clear signs of leaf litter shredding (Figure S4), suggesting large invertebrate species drove litter mass loss.

Decomposition is incredibly complex, highly context‐dependent and can change due to season (Mora‐Gómez et al., 2016), increased nutrient inputs (Wang et al., 2019) or shifting enzymatic expression associated with different stages of microbial succession (Hättenschwiler et al., 2005). In highly altered systems, macroinvertebrates are often the main drivers of decomposition, whereas bacteria (but not fungi) play a supplementary role (Tresch et al., 2019). As conditions become cooler and wetter, fungi may overtake invertebrates as the main drivers of decomposition, although they are far less efficient than invertebrates (Wu et al., 2021). Our results suggest a similar pattern as fungal diversity was highest during the winter sampling session and marginally linked to increasing decomposition rates. Conversely, fungi may also slow decomposition via antagonistic interactions between species with different life strategies (e.g. saprotrophic verse mycorrhizal fungi), which may explain why they were not significant drivers of decomposition in this study (Hättenschwiler et al., 2005).

Other than macroinvertebrate abundance, the only other significant direct driver of litter decomposition was increasing temperature during the winter sampling session. Although dependant on baseline temperatures, local increases in temperature can reduce soil moisture and are generally associated with slower litter decomposition as these conditions restrict enzymatic activity (Liu et al., 2022) and the abundance of invertebrate detritivores (Figueroa et al., 2021). These findings likely do not apply in our instance as our study sites were experiencing high rainfall and elevated moisture conditions 19 months post‐rewilding. Microbial activity is often limited by soil moisture, so as temperature rises in moist conditions, microbial and enzymatic activity can be sustained at high rates (Krishna & Mohan, 2017). Although the winter piecewise SEM did not establish a link between temperature—fungal diversity—decomposition, we cannot rule out that small increases in temperature at the site level may have interacted with elevated moisture to increase decomposition via an unmeasured variable such as bacterial diversity. Alternatively, temperature may have driven decomposition independently; a pattern that is found in terrestrial wood decomposition where temperature drives decomposition independent of invertebrate and microbial richness (Pietsch et al., 2019). Elevated moisture and thus favourable conditions across sites may have also maximised the functional efficiency of the decomposer community. This may explain both the reduced effect of ‘treatment’ and elevated decomposition rates during winter.

Given decomposition was largely driven by invertebrate detritivores in our instance, the next logical question is what species, if any, were driving this change. Our study system was dominated by several highly abundant invertebrate species. During the sampling session with the strongest treatment effect on decomposition (35 months post‐rewilding) three (17%) species accounted for 83.6% of total detritivore abundance (*Ommatoiulus moreleti*: 57.0%, *Ecnolagria* sp. 1: 14.8%, and *Calolampra* sp. 1: 11.7%). *Ommatoiulus moreleti* (Diplopoda: Julidae), *Ecnolagria* sp. 1 (Coleoptera: Tenebrionidae) and to a lesser extent *Calolampra* sp. 1 (Blattodea: Blaberidae), showed strong evidence (>90%) of an increase in abundances in the rewilding transplant site as compared to both the control and remnant treatments (Table S7). *Ommatoiulus moreleti* is a highly invasive species in southern Australia and is a major contributor to litter consumption, especially when litter is primed with white‐rot fungal species (Harrop‐Archibald et al., 2016). Little is known about the biology of the endemic *Calolampra* and *Ecnolagria*, although both were observed ingesting litter in the field. Macroinvertebrate detritivore abundance can rapidly (~3 years) reach that of nearby remnant forests during ex‐agricultural restoration (Stone et al., 2020). However, our results suggest again that the abundances of these species now exceed remnant levels. Increased nutrients can change community dynamics by facilitating highly competitive generalist species, making them numerically dominant in the community (Wild et al., 2022). Transplanting dominant species from likely nutrient‐poor remnant sites into nutrient‐rich restoration sites may have allowed these species to become highly abundant within rewilding transplant sites. Increased abundances may have also been facilitated by the comparatively species‐poor detritivore community that could be found in restoration sites relative to remnant sites, which would have allowed competitive species to become highly dominant in a nutrient‐rich environment (Cadotte et al., 2021).

## 5 CONCLUSIONS

Reduced functional capacity in restored sites is still a pervasive issue despite decades of monetary investment in restoration (Moreno‐Mateos et al., 2012; Rowland et al., 2009). We have shown that transplanting leaf litter can increase the efficiency of decomposition during ecological restoration. Although these are promising results, we note that litter transplants are unlikely to rewild social insects and may therefore be less effective where detritivorous termites are numerically dominant, and there are risks associated with litter transplants that need to be considered pre‐rewilding such as the spread of invasive species or pathogens (see Contos et al., 2021 for further discussion). If practitioners are particularly concerned about spreading invasive species, we suggest thorough monitoring of source sites prior to transplanting to properly assess the risk verse reward of invertebrate and microbial rewilding. Our results also suggest we should reassess our restoration targets during ex‐agricultural restoration to account for the higher productivity of restoration sites relative to remnants, and focus restoration efforts on restoring function rather than historical species communities (Parkhurst et al., 2022; Stone et al., 2020). We suggest monitoring soil nutrients and dynamics in conjunction with decomposition during future experiments to better understand site potential. We encourage further tests of our methodology in broader contexts (e.g. wetland/stream restoration) as full functional recovery is paramount for safeguarding communities and increasing the resilience of ecosystems that face continued degradation.

## AUTHOR CONTRIBUTIONS

**Peter Contos:** Conceptualization (lead); data curation (lead); formal analysis (lead); methodology (lead); visualization (lead); writing – original draft (lead); writing – review and editing (lead). **Nicholas P. Murphy:** Conceptualization (lead); formal analysis (equal); methodology (equal); writing – original draft (equal); writing – review and editing (equal). **Zachary J. Kayll:** Data curation (supporting); investigation (supporting); writing – review and editing (supporting). **Tamara Morgan:** Data curation (supporting); investigation (supporting); writing – review and editing (supporting). **Joshua J. Vido:** Data curation (supporting); formal analysis (supporting); methodology (supporting); writing – original draft (supporting); writing – review and editing (supporting). **Orsi Decker:** Data curation (supporting); formal analysis (supporting); methodology (supporting); writing – review and editing (supporting). **Heloise Gibb:** Conceptualization (lead); funding acquisition (lead); methodology (equal); project administration (lead); visualization (equal); writing – original draft (equal); writing – review and editing (equal).

## Supporting information


Appendix S1

## Data Availability

Data and code for analyses are available within Dryad at: https://doi.org/10.5061/dryad.ncjsxkt1r.
