# Supplementary material for: Rewilding soil and litter invertebrates and fungi increases decomposition rates and alters detritivore communities
Source: Ecol Evol. 2024 Mar 10;14(3):e11128. doi: 10.1002/ece3.11128 (PMC10925487; doi:10.1002/ece3.11128)
Supplement: Supplementary file 1 — Appendix S1 [file ECE3-14-e11128-s001.docx]

| Site | Treatment | Canopy tree species | Year planted | Size (ha) | Slope (%) | Elevation (m) |
| --- | --- | --- | --- | --- | --- | --- |
| 1 | Control | *Eucalyptus polyanthemos, E. goniocalyx, Acacia melanoxylon* | 2002 | 1.4 | 5 | 326 |
| 1 | Rewilding transplant | *Eucalyptus polyanthemos, E. goniocalyx, Acacia implexa, E. melliodora, E. rubida* | 2006 | 1.2 | 5 | 324 |
| 1 | Remnant (Gobur Nature Conservation Reserve) | *Eucalyptus radiata, E. obliqua* | N/A | 380 | 15 | 627 |
| 2 | Control | *Eucalyptus polyanthemos, E. radiata, Acacia dealbata, E. camphora* | 2006 | 1.1 | 45 | 308 |
| 2 | Rewilding transplant | *Eucalyptus polyanthemos, E. melliodora, E. radiata, Acacia dealbata, E. macrorhyncha, E. globulus* | 2006 | 1.2 | 50 | 324 |
| 2 | Remnant (Yarck Nature Conservation Reserve) | *Eucalyptus globulus, E. dives* | N/A | 515 | 35 | 394 |
| 3 | Control | *Eucalyptus globulus, E. albens, E. microcarpa* | 2005 | 1.1 | 10 | 387 |
| 3 | Rewilding transplant | *Eucalyptus melliodora, E. albens, E. microcarpa* | 2006 | 1.1 | 20 | 371 |
| 3 | Remnant (Dropmore G101 Bushland Reserve) | *Eucalyptus radiata, E. obliqua* | N/A | 38 | 10 | 636 |
| 4 | Control | *Eucalyptus goniocalyx, E. macrorhyncha, E. mearnsii* | 1998 | 0.5 | 0 | 465 |
| 4 | Rewilding transplant | *Acacia dealbata, Eucalyptus microcarpa, E. mearnsii, E. nitens* | 1999 | 0.7 | 0 | 450 |
| 4 | Remnant (Caveat Nature Conservation Reserve) | *Eucalyptus radiata, E. obliqua* | N/A | 123 | 25 | 623 |
| 5 | Control | *Eucalyptus polyanthemos, E. goniocalyx, Acacia implexa* | 2000 | 0.5 | 10 | 479 |
| 5 | Rewilding transplant | *Eucalyptus goniocalyx, Acacia implexa, E. camphora* | 2002 | 0.7 | 5 | 446 |
| 5 | Remnant (Dropmore G46 Bushland Reserve) | *Eucalyptus radiata, E. obliqua* | N/A | 122 | 5 | 646 |
| 6 | Control | *Eucalyptus globulus, E. viminalis* | 2006 | 2.9 | 5 | 386 |
| 6 | Rewilding transplant | *Eucalyptus globulus, E. viminalis* | 2006 | 5.3 | 15 | 425 |
| 6 | Remnant (Wallaby Gully Flora Reserve) | *Eucalyptus macrorhyncha, E. globulus* | N/A | 125 | 30 | 497 |

**Table S1:** Habitat characteristics for each site used in the study.

**
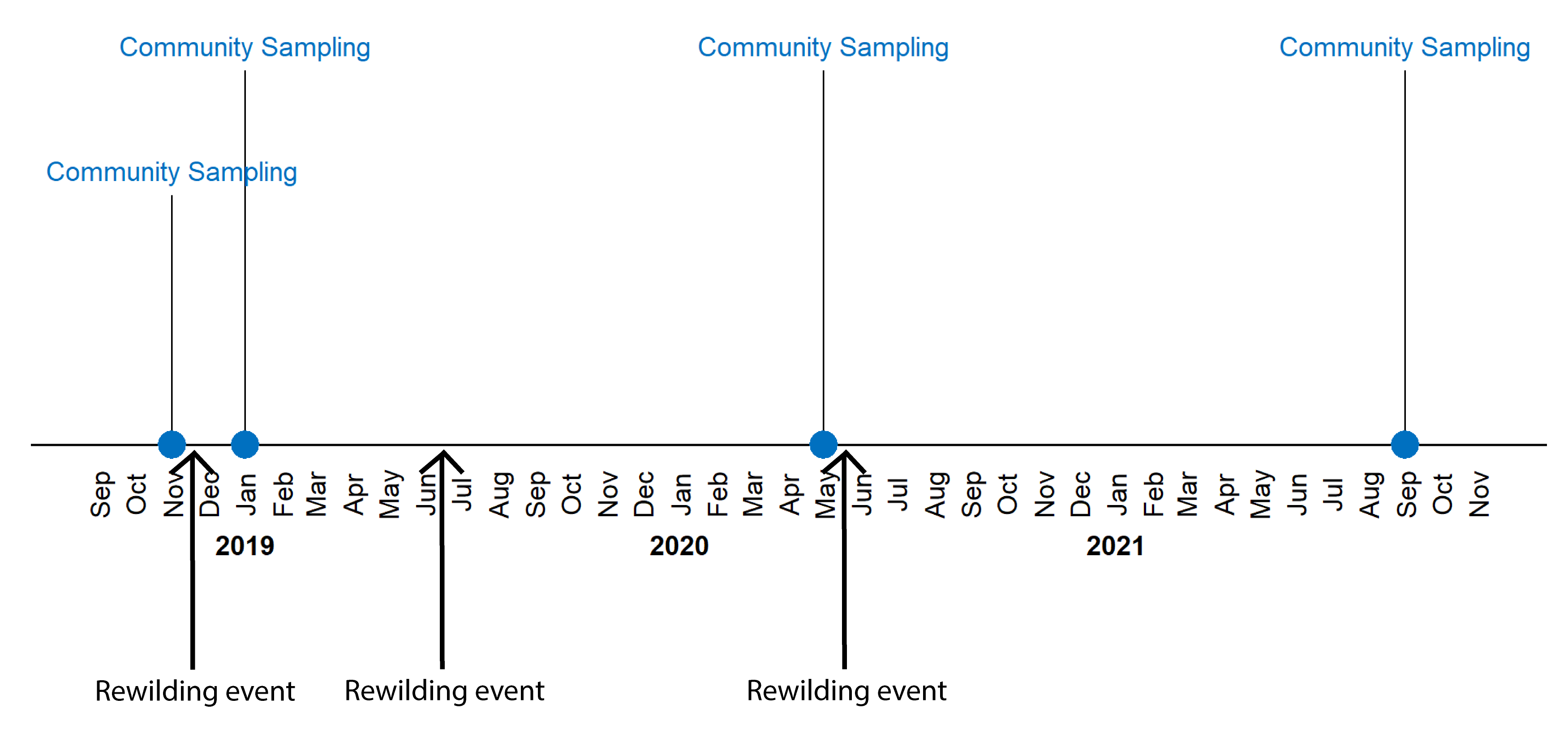
**

**Figure S1.** A timeline showing dates of community sampling and rewilding events.

**
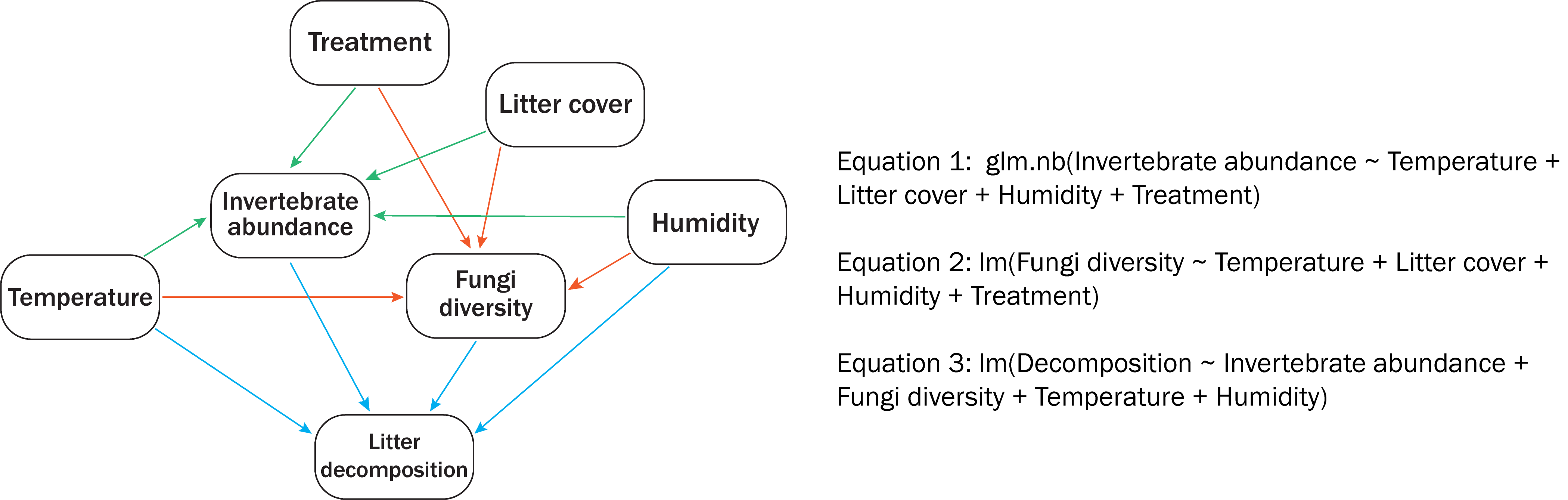
**

**Figure S2.** The three equations that composed each piecewise SEM. Equation 1 (green arrows) links abiotic variables and the experimental treatment to macroinvertebrate detritivore abundance and was run as a negative binomial generalised linear mixed model. Equation 2 (orange arrows) links abiotic variables to fungal saprotroph diversity and was run as a linear model. Equation 3 (blue arrows) links biotic and abiotic variables to the rate of leaf litter decomposition and was run as a linear model.

**Table S2.** Post-hoc comparisons for the main effect of ‘treatment’ in the GLMM decomposition model. ‘RT’ = rewilding transplant, ‘R’ = remnant, ‘C’ = control.

| **Contrast** | **Estimate** | ***t-*ratio** | ***P*** |
| --- | --- | --- | --- |
| RT vs C | 3.14 | 5.05 | <0.0001 |
| RT vs R | 2.00 | 3.21 | 0.005 |
| C vs R | -1.14 | -1.84 | 0.16 |

**Table S3.** Post-hoc outputs from the decomposition model across seasons. ‘RT’ = rewilding transplant, ‘R’ = remnant, ‘C’ = control.

| **Time** | **Pairing** | ***t*-ratio** | ***P*** |
| --- | --- | --- | --- |
| 3 months post | RT vs C | 2.04 | 0.11 |
|  | RT vs R | 0.90 | 0.64 |
|  | C vs R | -1.14 | 0.49 |
| 19 months post | RT vs C | 0.08 | 0.99 |
|  | RT vs R | 0.18 | 0.98 |
|  | C vs R | 0.11 | 0.99 |
| 35 months post | RT vs C | 6.63 | <0.0001 |
|  | RT vs R | 4.48 | <0.0001 |
|  | C vs R | -2.15 | 0.09 |

| **Species ID** | **Order** | **Pre-rewilding** | | | **3 months** | | | **19 months** | | | **35 months** | | |
| --- | --- | --- | --- | --- | --- | --- | --- | --- | --- | --- | --- | --- | --- |
|  |  | **C** | **RT** | **R** | **C** | **RT** | **R** | **C** | **RT** | **R** | **C** | **RT** | **R** |
| Talitridae sp. 1 | Amphipoda | 0 | 0 | 34 | 0 | 0 | 0 | 0 | 0 | 2 | 0 | 0 | 4 |
| Annelida sp. 1 | Annelida | 0 | 0 | 0 | 0 | 0 | 0 | 6 | 9 | 1 | 0 | 2 | 2 |
| *Calolampra* sp. 1 | Blattodea | 18 | 9 | 16 | 3 | 4 | 5 | 4 | 4 | 7 | 12 | 7 | 11 |
| Blattodea sp. 11 | Blattodea | 0 | 0 | 0 | 0 | 0 | 0 | 0 | 3 | 0 | 0 | 0 | 0 |
| Blattodea sp. 5 | Blattodea | 0 | 0 | 0 | 0 | 0 | 0 | 0 | 0 | 0 | 0 | 0 | 2 |
| Blattidae sp. 1 | Blattodea | 0 | 0 | 1 | 0 | 0 | 0 | 0 | 0 | 0 | 0 | 0 | 0 |
| Blattodea sp. 9 | Blattodea | 0 | 0 | 0 | 0 | 0 | 0 | 0 | 0 | 0 | 0 | 0 | 1 |
| Chrysomelidae sp. 1 | Coleoptera | 0 | 0 | 0 | 0 | 0 | 0 | 0 | 0 | 0 | 2 | 0 | 1 |
| Tenebrionidae sp. 1 | Coleoptera | 6 | 7 | 1 | 4 | 2 | 0 | 10 | 6 | 0 | 1 | 1 | 0 |
| Tenebrionidae sp. 7 | Coleoptera | 0 | 0 | 1 | 0 | 0 | 0 | 0 | 0 | 0 | 0 | 0 | 0 |
| Tenebrionidae sp. 8 | Coleoptera | 0 | 0 | 1 | 0 | 0 | 1 | 0 | 0 | 1 | 0 | 0 | 0 |
| Tenebrionidae sp. 2 | Coleoptera | 1 | 1 | 0 | 0 | 0 | 0 | 0 | 0 | 0 | 0 | 2 | 0 |
| Tenebrionidae sp. 3 | Coleoptera | 0 | 1 | 0 | 0 | 2 | 1 | 0 | 0 | 0 | 0 | 0 | 0 |
| Tenebrionidae sp. 4 | Coleoptera | 0 | 0 | 3 | 0 | 0 | 2 | 0 | 0 | 0 | 0 | 0 | 2 |
| Tenebrionidae sp. 9 | Coleoptera | 0 | 0 | 1 | 0 | 0 | 0 | 0 | 0 | 0 | 0 | 0 | 0 |
| Tenebrionidae sp. 10 | Coleoptera | 0 | 0 | 0 | 0 | 0 | 1 | 0 | 0 | 0 | 0 | 0 | 0 |
| *Ecnolagria* sp. 1 | Coleoptera | 7 | 10 | 6 | 0 | 0 | 0 | 3 | 5 | 9 | 4 | 25 | 9 |
| Tenebrionidae sp. 5 | Coleoptera | 0 | 0 | 0 | 0 | 0 | 0 | 0 | 0 | 0 | 0 | 1 | 1 |
| Tenebrionidae sp. 6 | Coleoptera | 2 | 1 | 0 | 0 | 1 | 0 | 0 | 0 | 0 | 0 | 0 | 0 |
| Paronellidae sp. 1 | Entomobryomorpha | 0 | 0 | 0 | 0 | 0 | 0 | 5 | 0 | 0 | 0 | 0 | 0 |
| Paradoxosomatidae sp. 4 | Diplopoda | 0 | 0 | 0 | 0 | 0 | 0 | 0 | 0 | 8 | 0 | 0 | 0 |
| Paradoxosomatidae sp. 5 | Diplopoda | 0 | 0 | 0 | 0 | 0 | 0 | 0 | 0 | 0 | 0 | 0 | 1 |
| Paradoxosomatidae sp. 6 | Diplopoda | 0 | 0 | 1 | 0 | 0 | 0 | 0 | 0 | 0 | 0 | 0 | 0 |
| Paradoxosomatidae sp. 1 | Diplopoda | 0 | 0 | 3 | 0 | 0 | 0 | 0 | 0 | 0 | 0 | 0 | 2 |
| Paradoxosomatidae sp. 2 | Diplopoda | 0 | 0 | 16 | 0 | 0 | 0 | 0 | 0 | 0 | 0 | 0 | 0 |
| Paradoxosomatidae sp. 3 | Diplopoda | 0 | 0 | 75 | 0 | 0 | 0 | 0 | 0 | 0 | 0 | 0 | 0 |
| Paradoxosomatidae sp. 7 | Diplopoda | 0 | 0 | 1 | 0 | 0 | 0 | 0 | 0 | 0 | 0 | 0 | 0 |
| Polyzoniida sp. 1 | Diplopoda | 0 | 0 | 0 | 0 | 0 | 0 | 0 | 0 | 0 | 0 | 2 | 1 |
| *Ommatoiulus moreletii* | Diplopoda | 333 | 503 | 36 | 14 | 64 | 3 | 31 | 28 | 15 | 39 | 81 | 28 |
| Philosciidae sp. 1 | Isopoda | 0 | 0 | 56 | 0 | 0 | 1 | 0 | 0 | 1 | 0 | 0 | 2 |
| Armadillidae sp. 1 | Isopoda | 0 | 0 | 8 | 0 | 0 | 0 | 0 | 0 | 0 | 0 | 0 | 0 |
| *Porcellio scaber* | Isopoda | 0 | 9 | 0 | 0 | 1 | 0 | 0 | 0 | 0 | 2 | 5 | 3 |
| Porcellionidae sp. 1 | Isopoda | 0 | 11 | 0 | 0 | 0 | 0 | 0 | 0 | 0 | 0 | 0 | 0 |
| Symphyla sp. 1 | Symphyla | 0 | 0 | 1 | 0 | 0 | 1 | 0 | 0 | 0 | 0 | 0 | 0 |
| Symphyla sp. 2 | Symphyla | 0 | 0 | 0 | 0 | 0 | 0 | 0 | 0 | 1 | 0 | 0 | 0 |
| Lepismatidae sp. 1 | Zygentoma | 1 | 0 | 0 | 0 | 1 | 0 | 0 | 0 | 0 | 0 | 0 | 0 |

**Table S4.** Raw abundances of invertebrate detritivore morphospecies/species found within each treatment at each time point. ‘RT’ = rewilding transplant, ‘R’ = remnant, ‘C’ = control.

**Table S5.** Post-hoc outputs from each of the combined post-rewilding GLMM models. ‘RT’ = rewilding transplant, ‘R’ = remnant, ‘C’ = control.

|  | **Invertebrate species richness** | | **Invertebrate abundance** | | **Fungi relative abundance** | | **Fungi diversity** | |
| --- | --- | --- | --- | --- | --- | --- | --- | --- |
| **Contrast** | ***Z* ratio** | ***P*** | ***Z* ratio** | ***P*** | ***Z* ratio** | ***P*** | ***Z* ratio** | ***P*** |
| RT vs C | 1.40 | 0.34 | 1.85 | 0.15 | 0.82 | 0.69 | 0.11 | 0.99 |
| RT vs R | -0.78 | 0.72 | 2.26 | 0.06 | 1.55 | 0.27 | 0.28 | 0.96 |
| C vs R | -2.11 | 0.09 | 0.42 | 0.91 | 0.77 | 0.72 | 0.18 | 0.98 |

**Table S6.** Proportion of variance (%) explained by each variable included in the invertebrate detritivore HMSC models.

| **Time** | **Site** | **Humidity** | **Temperature** | **Treatment** |
| --- | --- | --- | --- | --- |
| Pre-rewilding | 8.8 | 44.4 | 37.2 | 9.7 |
| 3 months | 6.2 | 42.4 | 28.2 | 23.3 |
| 19 months | 14.4 | 30.2 | 24.3 | 31.1 |
| 35 months | 5.5 | 23.2 | 17.4 | 53.9 |

| **Morphospecies/Species** | **RT vs C (Pre)** | **RT vs C (3 months)** | **RT vs C (19 months)** | **RT vs C (35 months)** | **RT vs R (Pre)** | **RT vs R (3 months)** | **RT vs R (19 months)** | **RT vs R (35 months)** |
| --- | --- | --- | --- | --- | --- | --- | --- | --- |
| Tenebrionidae *sp.* 6 | 0.00 |  |  |  | 0.76 |  |  |  |
| Tenebrionidae *sp*. 5 |  |  |  | 0.82 |  |  |  | 0.96 |
| Tenebrionidae *sp.* 4 | 0.78 |  |  |  | 0.00 |  |  |  |
| Tenebrionidae *sp*. 3 |  | 0.00 |  |  |  | 0.87 |  |  |
| Tenebrionidae *sp.* 2 | 0.00 |  |  | 0.83 | 0.76 |  |  | 0.97 |
| Tenebrionidae *sp.* 1 | 0.98 | -0.98 | -0.90 | 0.82 | 0.96 | 0.00 | 0.00 | 0.96 |
| *Ecnolagria* *sp*. 1 | 0.85 |  | -0.92 | 0.98 | 0.95 |  | 0.00 | 0.99 |
| Chrysomelidae *sp.* 1 |  |  |  | 0.77 |  |  |  | 0.98 |
| *Calolampra sp.* 1 | -0.88 | 0.00 | -0.75 | 0.80 | 1.00 | 0.00 | 0.00 | 1.00 |
| Philosciidae *sp*. 1 | 0.83 |  |  | 0.83 | 0.00 |  |  | 0.97 |
| *Porcellio scaber* |  |  |  | 0.99 |  |  |  | 1.00 |
| Talitridae *sp*. 1 | 0.84 |  | -0.81 | 0.84 | 0.00 |  | 0.00 | 0.97 |
| Paradoxosomatidae *sp*. 3 | 0.00 |  |  |  | 0.92 |  |  |  |
| Paradoxosomatidae *sp*. 2 | 0.80 |  | -0.86 |  | 0.00 |  | 0.00 |  |
| Paradoxosomatidae *sp*. 1 | 0.00 |  |  | 0.84 | 0.00 |  |  | 0.96 |
| *Ommatoiulus moreleti* | 0.00 | 0.00 | -0.96 | 0.93 | 0.87 | 0.91 | 0.00 | 0.99 |
| Polyzoniida *sp*. 1 |  |  |  | 0.85 |  |  |  | 0.99 |
| Annelida *sp*. 1 |  |  | -0.97 | 0.89 |  |  | 0.00 | 1.00 |

**Table S7.** Invertebrate detritivore morphospecies/species responses derived from each of the four HMSC models in the treatment pairings RT vs C (rewilding transplant vs control) and RT vs R (rewilding transplant vs remnant). Missing values indicate that the species was absent in that sampling session.

**Table S8.** Proportion of variance (%) explained by each variable included in the fungal saprotroph HMSC models.

| **Time** | **Site** | **Humidity** | **Temperature** | **Treatment** |
| --- | --- | --- | --- | --- |
| Pre-rewilding | 12.6 | 31.0 | 28.6 | 27.9 |
| 3 months | 12.0 | 22.8 | 22.4 | 42.8 |
| 19 months | 11.1 | 28.9 | 12.1 | 47.9 |
| 35 months | 9.8 | 31.7 | 19.9 | 38.6 |

**
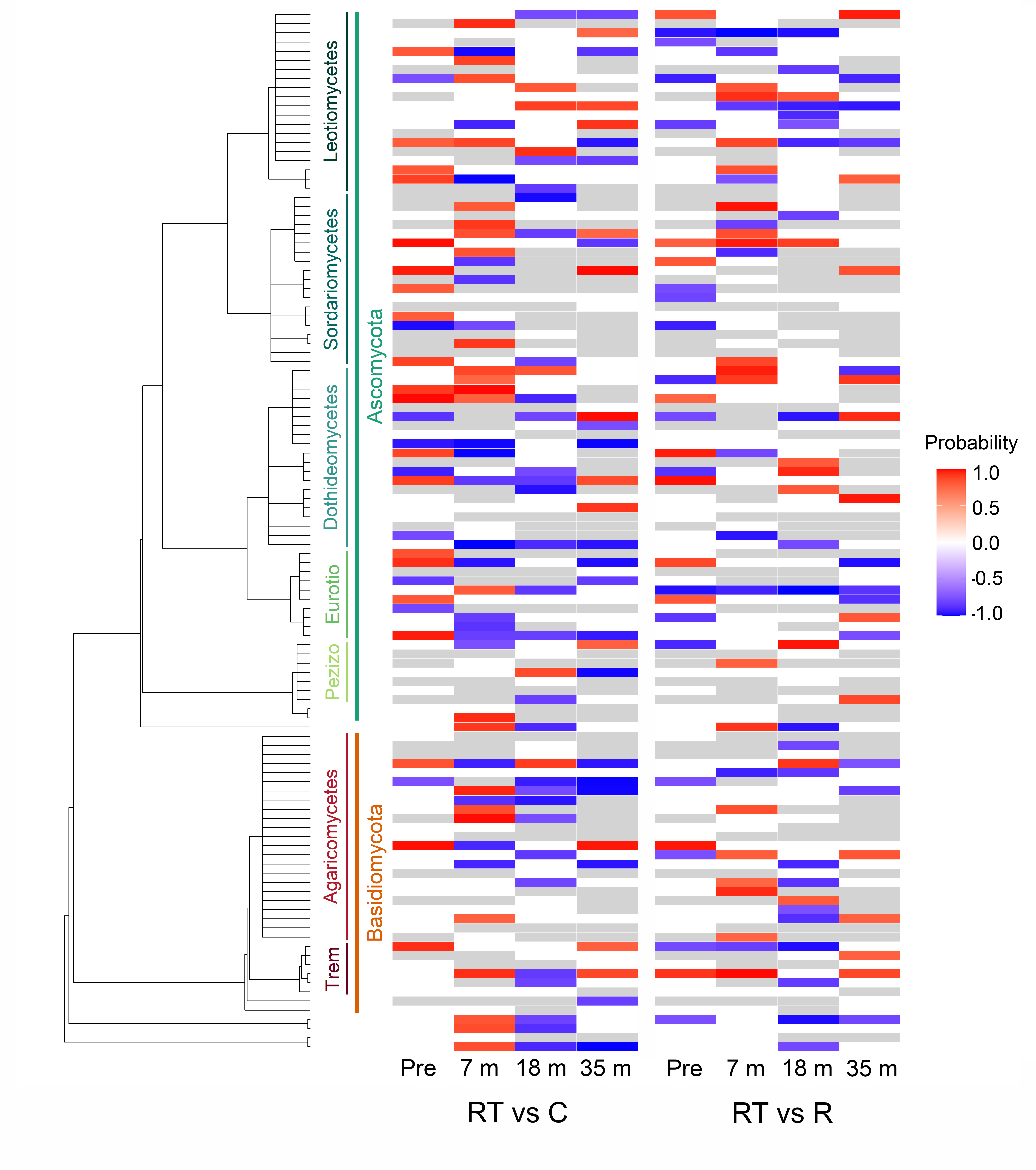
**

**Figure S3**. Saprotrophic fungal ASV responses to the treatment pairings RT vs C (rewilding transplant vs control) and RT vs R (rewilding transplant vs remnant) in the HMSC models across each sampling time point. Blue colours indicate negative responses (i.e., significantly less abundant in RT vs other treatments), red colours indicate positive responses with ≥0.75 posterior probability, white colours show ASVs that did not gain strong statistical support, and grey colours indicate a missing ASV in the sampling session. A phylogenetic tree shows taxonomic relationships between ASVs. Shortened labels: Eurotio = Eurotiomycetes, Pezizo = Pezizomycetes, Trem = Tremellomycetes.

**Table S9.** Saprotrophic fungal ASV responses derived from each of the four HMSC models in the treatment pairings RT vs C (rewilding transplant vs control) and RT vs R (rewilding transplant vs remnant). Missing values indicate that the species was absent in that sampling session.

| **ASV** | **Phylum** | **Class** | **RT vs C (Pre)** | **RT vs C (3 mo)** | **RT vs C (19 mo)** | **RT vs C (35 mo)** | **RT vs R (Pre)** | **RT vs R (3 mo)** | **RT vs R (19 mo)** | **RT vs R (35 mo)** |
| --- | --- | --- | --- | --- | --- | --- | --- | --- | --- | --- |
| ASV_111 | Ascomycota | Sordariomycetes |  |  |  | 0.00 |  |  |  | 0.00 |
| ASV_906 | Ascomycota | Sordariomycetes | 0.97 |  |  | 0.99 | 0.77 |  |  | 0.93 |
| ASV_807 | Ascomycota | Sordariomycetes | 0.81 |  |  |  | -0.79 |  |  |  |
| ASV_943 | Ascomycota | Sordariomycetes | 0.00 |  | 0.00 | 0.00 | 0.00 |  | -0.83 | 0.00 |
| ASV_706 | Ascomycota | Sordariomycetes | 0.88 |  | 0.00 |  | 0.00 |  | 0.00 |  |
| ASV_401 | Ascomycota | Sordariomycetes |  |  | -1.00 |  |  |  | -0.78 |  |
| ASV_366 | Ascomycota | Sordariomycetes |  |  | 0.00 |  |  |  | 0.00 |  |
| ASV_351 | Ascomycota | Sordariomycetes |  |  | 0.00 |  |  |  | 0.00 |  |
| ASV_186 | Ascomycota | Sordariomycetes |  |  | 0.00 |  |  |  | 0.00 |  |
| ASV_1164 | Ascomycota | Sordariomycetes | 0.00 | 0.81 |  |  | 0.00 | -0.92 |  |  |
| ASV_1159 | Ascomycota | Sordariomycetes | 0.00 | 0.76 | -0.92 |  | 0.00 | 0.00 | -0.84 |  |
| ASV_1152 | Ascomycota | Sordariomycetes |  | 0.89 |  |  |  | 0.00 |  |  |
| ASV_1050 | Ascomycota | Sordariomycetes | -0.93 | -0.87 |  |  | -0.78 | 0.00 |  |  |
| ASV_1006 | Ascomycota | Sordariomycetes | 0.00 | 0.00 | -0.76 | 0.87 | -0.78 | -0.98 | 0.00 | 0.91 |
| ASV_1000 | Ascomycota | Sordariomycetes | 0.00 | 0.87 | -0.89 | 0.00 | 0.00 | 0.86 | 0.00 | 0.83 |
| ASV_909 | Ascomycota | Sordariomycetes | 0.95 | 0.99 | -0.95 |  | 0.00 | 0.80 | 0.00 |  |
| ASV_881 | Ascomycota | Sordariomycetes |  | -0.89 |  |  |  | 0.00 |  |  |
| ASV_825 | Ascomycota | Sordariomycetes | 0.00 | 0.00 | -0.94 | 0.00 | -0.80 | -0.83 | -0.86 | 0.00 |
| ASV_775 | Ascomycota | Sordariomycetes | 0.80 | 0.00 |  |  | 0.00 | 0.00 |  |  |
| ASV_750 | Ascomycota | Sordariomycetes | 0.00 | -0.87 |  |  | 0.80 | 0.00 |  |  |
| ASV_720 | Ascomycota | Sordariomycetes | 0.00 | 0.81 | 0.00 | 0.89 | 0.00 | 0.00 | 0.00 | 0.80 |
| ASV_705 | Ascomycota | Sordariomycetes | 0.94 | 0.97 | 0.94 | 0.99 | 0.00 | 0.00 | 0.00 | 0.92 |
| ASV_677 | Ascomycota | Sordariomycetes |  | 1.00 | 0.96 |  |  | 0.00 | 0.00 |  |
| ASV_574 | Ascomycota | Sordariomycetes | 0.00 | 0.00 |  |  | 0.00 | 0.00 |  |  |
| ASV_552 | Ascomycota | Sordariomycetes | 0.87 | 0.91 | 0.00 | 1.00 | 0.88 | 0.89 | 0.00 | 0.99 |
| ASV_462 | Ascomycota | Sordariomycetes | 0.87 | 0.84 | -1.00 |  | 0.00 | 0.87 | -0.75 |  |
| ASV_396 | Ascomycota | Sordariomycetes | 0.99 | 0.80 | 0.00 | -0.78 | 0.83 | 0.97 | 0.85 | 0.00 |
| ASV_393 | Ascomycota | Sordariomycetes | -0.76 | -0.97 | -0.82 | 0.00 | 0.00 | -0.82 | 0.00 | -0.77 |
| ASV_369 | Ascomycota | Sordariomycetes |  | 0.94 |  |  |  | -0.81 |  |  |
| ASV_352 | Ascomycota | Sordariomycetes |  | 0.89 | 0.00 |  |  | 0.97 | 0.00 |  |
| ASV_331 | Ascomycota | Sordariomycetes | -0.98 | -0.80 |  |  | -0.92 | 0.00 |  |  |
| ASV_322 | Ascomycota | Sordariomycetes | 0.86 | 0.00 | -0.79 | 0.00 | 0.93 | 0.00 | -0.88 | 0.00 |
| ASV_265 | Ascomycota | Sordariomycetes |  | 0.76 | -0.94 | 0.00 |  | -0.80 | -0.97 | 0.00 |
| ASV_248 | Ascomycota | Sordariomycetes | 0.00 | 0.99 | 0.78 |  | 0.00 | 0.79 | 0.98 |  |
| ASV_246 | Ascomycota | Sordariomycetes | 0.00 | 0.00 | 0.00 | 0.00 | -0.87 | 0.79 | 0.00 | 0.00 |
| ASV_140 | Ascomycota | Sordariomycetes | 0.88 | 0.00 | -0.83 | 0.00 | 0.00 | 0.88 | 0.00 | 0.00 |
| ASV_114 | Ascomycota | Sordariomycetes | 0.00 | 0.92 |  |  | 0.00 | 0.00 |  |  |
| ASV_93 | Ascomycota | Sordariomycetes | -0.87 | 0.00 | 0.00 | 0.00 | -0.81 | 0.00 | 0.00 | 0.00 |
| ASV_1163 | Ascomycota | Leotiomycetes |  |  | -0.86 |  |  |  | 0.00 |  |
| ASV_1132 | Ascomycota | Leotiomycetes | 0.00 |  | -0.86 | -0.86 | 0.00 |  | 0.00 | 0.00 |
| ASV_1034 | Ascomycota | Leotiomycetes |  |  | 0.00 |  |  |  | 0.00 |  |
| ASV_891 | Ascomycota | Leotiomycetes | 0.00 |  | 0.00 | 0.00 | 0.00 |  | 0.00 | 0.00 |
| ASV_347 | Ascomycota | Leotiomycetes |  |  | 0.97 |  |  |  | 0.00 |  |
| ASV_108 | Ascomycota | Leotiomycetes | 0.00 |  | -0.84 |  | 0.00 |  | 0.00 |  |
| ASV_82 | Ascomycota | Leotiomycetes |  |  | 0.00 |  |  |  | -0.79 |  |
| ASV_1171 | Ascomycota | Leotiomycetes | -0.75 | 0.91 | -0.88 |  | 0.00 | 0.00 | 0.00 |  |
| ASV_1116 | Ascomycota | Leotiomycetes | 0.00 | 0.00 | 0.00 | 0.83 | -0.97 | -1.00 | -0.98 | 0.83 |
| ASV_900 | Ascomycota | Leotiomycetes | 0.79 | 0.76 | 0.00 | 0.00 | 0.00 | 0.86 | 0.00 | 0.00 |
| ASV_768 | Ascomycota | Leotiomycetes | 0.00 | 0.00 | 0.00 | 0.00 | 0.00 | 0.00 | -0.88 | 0.00 |
| ASV_717 | Ascomycota | Leotiomycetes |  | 0.00 | 0.00 |  |  | 0.00 | 0.00 |  |
| ASV_694 | Ascomycota | Leotiomycetes | -0.78 | 0.85 | 0.00 | 0.00 | -0.91 | 0.79 | 0.00 | -0.78 |
| ASV_618 | Ascomycota | Leotiomycetes | 0.00 | 0.00 | 0.00 |  | 0.00 | 0.00 | 0.00 |  |
| ASV_595 | Ascomycota | Leotiomycetes | 0.99 | 0.95 | 0.82 | 0.00 | 0.87 | 0.00 | 0.00 | 0.00 |
| ASV_590 | Ascomycota | Leotiomycetes | 0.00 | 0.00 | 0.86 |  | 0.00 | 0.83 | 0.00 |  |
| ASV_577 | Ascomycota | Leotiomycetes |  | 0.00 | 0.82 | 0.00 |  | 0.86 | 0.87 | 0.00 |
| ASV_576 | Ascomycota | Leotiomycetes | 0.90 | -0.99 | 0.82 | 0.88 | 0.00 | -0.84 | 0.84 | 0.89 |
| ASV_459 | Ascomycota | Leotiomycetes | 0.77 | 0.92 | 0.00 | -0.94 | 0.00 | 0.91 | -0.95 | -0.78 |
| ASV_403 | Ascomycota | Leotiomycetes | -0.85 | 0.78 |  |  | 0.88 | 0.80 |  |  |
| ASV_386 | Ascomycota | Leotiomycetes | 0.82 | -0.99 | 0.00 | -0.83 | 0.00 | -0.93 | 0.00 | 0.00 |
| ASV_372 | Ascomycota | Leotiomycetes |  | 0.00 |  |  |  | 0.98 |  |  |
| ASV_296 | Ascomycota | Leotiomycetes | 0.00 | 0.00 | 0.79 | 0.76 | 0.00 | -0.88 | -0.80 | -0.81 |
| ASV_158 | Ascomycota | Leotiomycetes |  | 0.00 | 0.00 |  |  | 0.00 | 0.00 |  |
| ASV_128 | Ascomycota | Leotiomycetes | 0.00 | -0.94 | 0.00 | 0.86 | -0.83 | 0.00 | -0.76 | 0.76 |
| ASV_116 | Ascomycota | Leotiomycetes | 0.00 | 0.00 | 0.00 | 0.76 | 0.00 | 0.00 | -0.88 | 0.82 |
| ASV_42 | Ascomycota | Leotiomycetes |  | 0.96 |  |  |  | 0.80 |  |  |
| ASV_3 | Ascomycota | Leotiomycetes | 0.00 | 0.00 | -0.82 | 0.00 | 0.90 | 0.00 | 0.00 | 0.93 |
| ASV_529 | Ascomycota | Dothideomycetes |  |  |  | -0.93 |  |  |  | 0.00 |
| ASV_502 | Ascomycota | Dothideomycetes |  |  |  | 0.77 |  |  |  | 0.00 |
| ASV_451 | Ascomycota | Dothideomycetes |  |  |  | 0.00 |  |  |  | 0.00 |
| ASV_1168 | Ascomycota | Dothideomycetes | 0.00 |  |  |  | -0.77 |  |  |  |
| ASV_584 | Ascomycota | Dothideomycetes | 0.00 |  |  |  | 0.00 |  |  |  |
| ASV_1134 | Ascomycota | Dothideomycetes |  |  | -0.89 |  |  |  | 0.00 |  |
| ASV_1124 | Ascomycota | Dothideomycetes |  |  | 0.00 | -0.87 |  |  | 0.00 | 0.00 |
| ASV_959 | Ascomycota | Dothideomycetes |  |  | -0.99 |  |  |  | 0.00 |  |
| ASV_911 | Ascomycota | Dothideomycetes |  |  | 0.00 |  |  |  | 0.00 |  |
| ASV_653 | Ascomycota | Dothideomycetes | 0.00 |  | 0.00 | 0.86 | 0.00 |  | 0.00 | 0.99 |
| ASV_479 | Ascomycota | Dothideomycetes |  |  | -0.94 |  |  |  | 0.00 |  |
| ASV_308 | Ascomycota | Dothideomycetes |  |  | -1.00 | -0.94 |  |  | 0.00 | 0.00 |
| ASV_172 | Ascomycota | Dothideomycetes | -0.92 |  | -0.85 | 1.00 | -0.83 |  | -0.97 | 0.98 |
| ASV_1093 | Ascomycota | Dothideomycetes | 0.84 | -0.88 | -0.87 | 0.89 | 0.98 | 0.00 | 0.00 | 0.00 |
| ASV_1078 | Ascomycota | Dothideomycetes | 0.92 | 0.99 | 0.00 |  | 0.00 | 0.00 | 0.00 |  |
| ASV_1058 | Ascomycota | Dothideomycetes | 1.00 | 0.81 | -0.95 |  | 0.80 | 0.00 | 0.00 |  |
| ASV_1041 | Ascomycota | Dothideomycetes | 0.00 | 0.00 |  |  | 0.00 | -0.95 |  |  |
| ASV_967 | Ascomycota | Dothideomycetes | 0.00 | 0.00 | 0.00 | 0.93 | 0.00 | 0.00 | 0.00 | 0.80 |
| ASV_957 | Ascomycota | Dothideomycetes | 0.00 | -1.00 | -0.97 | -0.99 | 0.00 | 0.00 | -0.88 | 0.00 |
| ASV_933 | Ascomycota | Dothideomycetes | -0.91 | 0.00 | 0.00 | 0.80 | -0.79 | 0.00 | 0.00 | 0.78 |
| ASV_866 | Ascomycota | Dothideomycetes | 0.00 | -0.97 | -0.99 |  | 0.00 | 0.00 | 0.00 |  |
| ASV_841 | Ascomycota | Dothideomycetes |  | 0.00 | -0.77 |  |  | 0.00 | 0.00 |  |
| ASV_746 | Ascomycota | Dothideomycetes | -0.99 | -0.99 | 0.00 | -1.00 | 0.00 | 0.00 | 0.76 | -0.79 |
| ASV_629 | Ascomycota | Dothideomycetes | 0.00 | 0.78 | 0.91 | 0.00 | 0.00 | 0.99 | 0.00 | 0.00 |
| ASV_601 | Ascomycota | Dothideomycetes |  | 0.75 |  |  |  | 0.00 |  |  |
| ASV_539 | Ascomycota | Dothideomycetes | 0.00 | 0.00 | 0.00 | 0.00 | 0.00 | -0.88 | 0.80 | 0.00 |
| ASV_513 | Ascomycota | Dothideomycetes | -0.82 | 0.96 | 0.00 | -0.96 | 0.00 | 0.97 | 0.00 | 0.00 |
| ASV_499 | Ascomycota | Dothideomycetes | 0.78 | 0.98 | 0.00 | -0.78 | 0.00 | 0.00 | 0.00 | 0.00 |
| ASV_480 | Ascomycota | Dothideomycetes | 0.84 | -0.99 | 0.00 |  | 0.96 | -0.86 | 0.00 |  |
| ASV_379 | Ascomycota | Dothideomycetes |  | -0.96 | 0.77 | 0.76 |  | -0.90 | 0.00 | 0.79 |
| ASV_354 | Ascomycota | Dothideomycetes | 0.00 | 0.83 | 0.00 | 0.00 | -0.86 | 0.91 | 0.00 | 0.87 |
| ASV_333 | Ascomycota | Dothideomycetes | 0.00 | 0.00 | 0.00 | 0.99 | 0.00 | 0.00 | 0.00 | 0.91 |
| ASV_279 | Ascomycota | Dothideomycetes |  | 0.00 | 0.00 | 0.00 |  | 0.00 | 0.00 | 0.00 |
| ASV_238 | Ascomycota | Dothideomycetes | 0.00 | 0.00 | -0.93 | 0.98 | 0.00 | 0.00 | -0.85 | 0.97 |
| ASV_235 | Ascomycota | Dothideomycetes | 0.77 | 0.80 | -0.81 | 0.86 | 0.00 | 0.00 | -0.85 | 0.79 |
| ASV_198 | Ascomycota | Dothideomycetes | -0.95 | 0.00 | 0.00 |  | -0.79 | 0.00 | 0.95 |  |
| ASV_183 | Ascomycota | Dothideomycetes |  | 0.00 |  |  |  | 0.00 |  |  |
| ASV_135 | Ascomycota | Dothideomycetes | 0.00 | 0.78 |  |  | 0.00 | 0.00 |  |  |
| ASV_464 | Ascomycota | Eurotiomycetes |  |  |  | 0.00 |  |  |  | 0.00 |
| ASV_1166 | Ascomycota | Eurotiomycetes | -0.84 |  |  | -0.90 | 0.00 |  |  | 0.00 |
| ASV_809 | Ascomycota | Eurotiomycetes | -0.77 |  |  |  | 0.00 |  |  |  |
| ASV_800 | Ascomycota | Eurotiomycetes | 0.00 |  |  |  | 0.00 |  |  |  |
| ASV_1157 | Ascomycota | Eurotiomycetes | 0.00 | 0.81 | -0.90 | 0.00 | -0.97 | -0.90 | -1.00 | 0.00 |
| ASV_1079 | Ascomycota | Eurotiomycetes | 0.00 | 0.00 | -0.86 | -0.86 | 0.88 | 0.98 | 0.00 | 0.00 |
| ASV_1039 | Ascomycota | Eurotiomycetes | 0.75 | 0.00 | 0.80 | 0.00 | 0.81 | 0.00 | 0.79 | 0.00 |
| ASV_890 | Ascomycota | Eurotiomycetes | -0.82 | 0.83 |  |  | 0.00 | 0.75 |  |  |
| ASV_731 | Ascomycota | Eurotiomycetes | 0.00 | 0.00 | 0.00 | 0.00 | 0.00 | 0.00 | -0.86 | 0.00 |
| ASV_510 | Ascomycota | Eurotiomycetes | 0.00 | 0.99 | 0.00 | -1.00 | 0.86 | 0.99 | 0.00 | -0.94 |
| ASV_419 | Ascomycota | Eurotiomycetes | 0.00 | 0.00 | 0.00 | 0.88 | -0.87 | 0.00 | 0.00 | 0.92 |
| ASV_320 | Ascomycota | Eurotiomycetes | 0.00 | -0.86 |  | 0.00 | 0.00 | 0.00 |  | 0.00 |
| ASV_225 | Ascomycota | Eurotiomycetes | 0.00 | 0.86 | 0.00 | 0.98 | 0.78 | 0.97 | 0.00 | 0.98 |
| ASV_51 | Ascomycota | Eurotiomycetes | 0.98 | -0.81 | -0.85 | -0.96 | 0.00 | -0.78 | 0.00 | -0.86 |
| ASV_10 | Ascomycota | Eurotiomycetes | 0.93 | -0.97 | 0.77 | -0.98 | 0.88 | 0.00 | 0.00 | -0.98 |
| ASV_151 | Ascomycota | Pezizomycetes | 0.00 |  |  |  | 0.00 |  |  |  |
| ASV_805 | Ascomycota | Pezizomycetes |  |  | -0.85 | 0.00 |  |  | -0.78 | 0.86 |
| ASV_455 | Ascomycota | Pezizomycetes |  |  | 0.00 |  |  |  | 0.00 |  |
| ASV_200 | Ascomycota | Pezizomycetes |  |  | -0.85 |  |  |  | 0.00 |  |
| ASV_168 | Ascomycota | Pezizomycetes |  |  | -0.76 |  |  |  | -0.76 |  |
| ASV_755 | Ascomycota | Pezizomycetes | 0.00 | 1.00 |  |  | 0.00 | 0.95 |  |  |
| ASV_566 | Ascomycota | Pezizomycetes | 0.00 | 0.00 | 0.82 | -1.00 | 0.00 | 0.00 | 0.00 | -0.78 |
| ASV_413 | Ascomycota | Pezizomycetes | 0.00 | 0.00 | 0.85 | 0.82 | -0.85 | 0.00 | 1.00 | 0.00 |
| ASV_76 | Ascomycota | Pezizomycetes |  | 0.79 |  |  |  | 0.77 |  |  |
| ASV_932 | Ascomycota | Geoglossomycetes | 0.00 | 0.00 |  |  | 0.00 | 0.00 |  |  |
| ASV_60 | Ascomycota | Geoglossomycetes | 0.00 | 0.95 |  |  | 0.00 | 0.00 |  |  |
| ASV_1063 | Ascomycota | Orbiliomycetes | -0.80 | 0.00 | 0.00 |  | 0.00 | 0.00 | 0.00 |  |
| ASV_993 | Ascomycota | Archaeorhizomycetes | 0.00 | 0.87 | -0.97 | 0.00 | 0.00 | 0.87 | -0.99 | 0.00 |
| ASV_899 | Basidiomycota | Agaricomycetes | 0.00 |  |  |  | 0.00 |  |  |  |
| ASV_564 | Basidiomycota | Agaricomycetes | 0.00 |  |  |  | 0.00 |  |  |  |
| ASV_165 | Basidiomycota | Agaricomycetes | 0.00 |  |  |  | 0.00 |  |  |  |
| ASV_1109 | Basidiomycota | Agaricomycetes |  |  | 0.00 |  |  |  | 0.00 |  |
| ASV_1091 | Basidiomycota | Agaricomycetes |  |  | 0.00 | 0.00 |  |  | 0.00 | 0.00 |
| ASV_868 | Basidiomycota | Agaricomycetes | -0.83 |  | -0.96 | -0.99 | -0.80 |  | 0.00 | 0.00 |
| ASV_827 | Basidiomycota | Agaricomycetes |  |  | 0.00 |  |  |  | 0.77 |  |
| ASV_739 | Basidiomycota | Agaricomycetes |  |  | 0.00 |  |  |  | -0.83 |  |
| ASV_580 | Basidiomycota | Agaricomycetes |  |  | 0.00 |  |  |  | 0.00 |  |
| ASV_281 | Basidiomycota | Agaricomycetes |  |  | -0.96 |  |  |  | 0.00 |  |
| ASV_1046 | Basidiomycota | Agaricomycetes | 0.00 | -0.76 | 0.76 | 0.00 | 0.00 | -0.94 | -0.83 | 0.00 |
| ASV_1035 | Basidiomycota | Agaricomycetes | 0.00 | 0.00 |  |  | 0.00 | 0.91 |  |  |
| ASV_1021 | Basidiomycota | Agaricomycetes | 0.00 | 1.00 | 0.00 | 0.00 | 0.82 | 0.82 | 0.00 | 0.00 |
| ASV_1011 | Basidiomycota | Agaricomycetes | 0.00 | 0.98 | -0.83 | -0.99 | 0.00 | 0.78 | 0.00 | -0.88 |
| ASV_942 | Basidiomycota | Agaricomycetes |  | 0.99 | -0.88 |  |  | 0.76 | -0.79 |  |
| ASV_895 | Basidiomycota | Agaricomycetes |  | 0.00 |  |  |  | -0.77 |  |  |
| ASV_852 | Basidiomycota | Agaricomycetes | 0.79 | -0.84 | 0.90 | 0.00 | 0.00 | 0.00 | 0.84 | 0.00 |
| ASV_789 | Basidiomycota | Agaricomycetes | -0.79 | 0.00 | -0.95 | -0.97 | 0.00 | 0.79 | -0.94 | 0.78 |
| ASV_742 | Basidiomycota | Agaricomycetes | -0.99 | -0.96 | -0.99 |  | -0.79 | 0.00 | 0.00 |  |
| ASV_701 | Basidiomycota | Agaricomycetes | 0.00 | 0.00 | -0.93 | 0.00 | -0.78 | 0.00 | 0.00 | 0.86 |
| ASV_609 | Basidiomycota | Agaricomycetes | 0.00 | 0.00 | 0.00 | 0.00 | 0.00 | -0.78 | -0.96 | 0.00 |
| ASV_563 | Basidiomycota | Agaricomycetes |  | 0.00 |  |  |  | 0.80 |  |  |
| ASV_554 | Basidiomycota | Agaricomycetes | 0.00 | -0.91 | -0.77 | -0.98 | 0.00 | 0.00 | -0.94 | 0.00 |
| ASV_537 | Basidiomycota | Agaricomycetes | 0.76 | 0.00 | 0.00 |  | 0.00 | 0.00 | 0.00 |  |
| ASV_498 | Basidiomycota | Agaricomycetes |  | 0.00 |  |  |  | 0.00 |  |  |
| ASV_473 | Basidiomycota | Agaricomycetes |  | -0.99 |  |  |  | -0.87 |  |  |
| ASV_463 | Basidiomycota | Agaricomycetes |  | 0.00 |  |  |  | 0.00 |  |  |
| ASV_427 | Basidiomycota | Agaricomycetes | -0.98 | -0.89 | 0.85 |  | -0.88 | -0.77 | 0.00 |  |
| ASV_268 | Basidiomycota | Agaricomycetes | 0.00 | -0.91 | -0.99 |  | 0.00 | 0.00 | 0.00 |  |
| ASV_185 | Basidiomycota | Agaricomycetes | 0.00 | 0.93 |  |  | 0.00 | 0.92 |  |  |
| ASV_133 | Basidiomycota | Agaricomycetes | 0.83 | -0.94 | 0.96 | -0.98 | 0.00 | 0.00 | 0.97 | -0.85 |
| ASV_66 | Basidiomycota | Agaricomycetes | 0.00 | 0.80 | 0.00 | 0.00 | 0.00 | 0.00 | -0.91 | 0.00 |
| ASV_56 | Basidiomycota | Agaricomycetes | 0.00 | 0.00 | -0.77 | 0.00 | 0.00 | 0.00 | -0.80 | 0.00 |
| ASV_41 | Basidiomycota | Agaricomycetes | 0.00 | 0.00 |  |  | 0.00 | 0.00 |  |  |
| ASV_21 | Basidiomycota | Agaricomycetes |  | 0.96 |  |  |  | 0.00 |  |  |
| ASV_14 | Basidiomycota | Agaricomycetes | 0.99 | -0.90 | 0.00 | 0.99 | 0.99 | 0.00 | 0.00 | 0.91 |
| ASV_1096 | Basidiomycota | Tremellomycetes |  |  |  | -0.78 |  |  |  | -0.81 |
| ASV_1173 | Basidiomycota | Tremellomycetes | 0.00 |  |  | 0.00 | 0.00 |  |  | 0.00 |
| ASV_1140 | Basidiomycota | Tremellomycetes | 0.76 |  | 0.00 | 0.87 | 0.78 |  | 0.00 | 0.81 |
| ASV_732 | Basidiomycota | Tremellomycetes | 0.00 |  | -0.84 | 0.00 | 0.00 |  | -0.90 | 0.00 |
| ASV_242 | Basidiomycota | Tremellomycetes |  |  | -0.98 |  |  |  | -0.80 |  |
| ASV_182 | Basidiomycota | Tremellomycetes |  |  | 0.00 | 0.00 |  |  | 0.00 | 0.83 |
| ASV_919 | Basidiomycota | Tremellomycetes | 0.00 | 0.97 | -0.87 | 0.86 | 0.95 | 1.00 | 0.00 | 0.90 |
| ASV_769 | Basidiomycota | Tremellomycetes |  | 0.00 | 0.00 | 0.00 |  | 0.00 | -0.79 | 0.81 |
| ASV_442 | Basidiomycota | Tremellomycetes | 0.90 | 0.00 | 0.00 | 0.80 | -0.80 | -0.81 | -0.98 | 0.00 |
| ASV_319 | Basidiomycota | Tremellomycetes | 0.85 | -0.94 | 0.00 | -0.79 | 0.85 | -0.77 | 0.00 | 0.00 |
| ASV_84 | Basidiomycota | Tremellomycetes | 0.00 | 0.00 | 0.00 |  | 0.00 | 0.00 | 0.00 |  |
| ASV_888 | Basidiomycota | Microbotryomycetes |  |  |  | -0.81 |  |  |  | 0.00 |
| ASV_27 | Basidiomycota | Microbotryomycetes |  |  |  | 0.00 |  |  |  | 0.00 |
| ASV_904 | Basidiomycota | Microbotryomycetes |  |  | -0.95 |  |  |  | 0.00 |  |
| ASV_767 | Basidiomycota | Microbotryomycetes | 0.00 | -0.80 | 0.84 |  | 0.00 | -0.88 | 0.00 |  |
| ASV_689 | Basidiomycota | Microbotryomycetes |  | 0.99 |  |  |  | 0.76 |  |  |
| ASV_152 | Basidiomycota | Microbotryomycetes | 0.00 | -0.85 | -0.91 |  | 0.93 | 0.00 | 0.00 |  |
| ASV_753 | Basidiomycota | Cystobasidiomycetes | 0.00 |  |  |  | 0.00 |  |  |  |
| ASV_748 | Basidiomycota | Atractiellomycetes |  | 0.00 |  |  |  | 0.00 |  |  |
| ASV_662 | Basidiomycota | Agaricostilbomycetes |  |  | 0.92 |  |  |  | 0.00 |  |
| ASV_603 | Basidiomycota | Geminibasidiomycetes | 0.00 | 0.00 |  | 0.00 | 0.00 | 0.00 |  | 0.00 |
| ASV_314 | Mucoromycota | Umbelopsidomycetes | 0.00 | 0.83 | -0.88 | 0.00 | 0.00 | 0.00 | -0.99 | 0.00 |
| ASV_187 | Mortierellomycota | Mortierellomycetes | 0.00 | 0.88 | -0.90 | 0.00 | 0.00 | 0.00 | 0.00 | 0.00 |
| ASV_964 | Chytridiomycota | Rhizophlyctidomycetes | 0.00 | 0.00 |  |  | 0.00 | 0.00 |  |  |
| ASV_406 | Chytridiomycota | Rhizophlyctidomycetes | 0.00 | 0.82 | -0.94 | -0.99 | 0.00 | 0.00 | -0.84 | -0.78 |
| ASV_233 | Chytridiomycota | Spizellomycetes |  | 0.00 |  |  |  | 0.00 |  |  |
| ASV_69 | Chytridiomycota | Spizellomycetes | -0.96 | 0.92 |  |  | -0.81 | 0.00 |  |  |

**Table S10.** Piecewise SEM results from each sampling session for all relationships that were included in the models. ‘NAs’ indicate pathways that were removed due to high VIF values (> 3). All pathways that were removed were not significant.

| **Response** | **Predictor** | **3 months post-rewilding** | | **19 months post-rewilding** | | **35 months post-rewilding** | |
| --- | --- | --- | --- | --- | --- | --- | --- |
|  |  | ***P*** | **Standard estimate** | ***P*** | **Standard estimate** | ***P*** | **Standard estimate** |
| Fungi diversity | Temperature | NA | NA | 0.67 | 0.14 | 0.20 | 0.49 |
| Fungi diversity | Litter cover | 0.98 | 0.01 | 0.51 | 0.29 | 0.84 | -0.07 |
| Fungi diversity | Humidity | 0.32 | 0.26 | 0.84 | -0.07 | 0.61 | 0.17 |
| Fungi diversity | Treatment | 0.31 | 0.28 | 0.66 | -0.13 | 0.70 | 0.14 |
| Macroinvertebrate abundance | Temperature | NA | NA | 0.04 | -0.14 | 0.02 | 0.21 |
| Macroinvertebrate abundance | Litter cover | 0.23 | 0.30 | 0.11 | -0.09 | 0.01 | 0.25 |
| Macroinvertebrate abundance | Humidity | 0.26 | 0.28 | 0.053 | -0.12 | 0.02 | -0.21 |
| Macroinvertebrate abundance | Treatment | 0.33 | -0.23 | 0.07 | -0.09 | 0.03 | 0.19 |
| Decomposition | Macroinvertebrate abundance | 0.01 | 0.61 | 0.78 | 0.07 | 0.09 | 0.60 |
| Decomposition | Temperature | 0.57 | -0.16 | 0.04 | 0.65 | 0.42 | -0.27 |
| Decomposition | Humidity | 0.48 | -0.19 | 0.27 | 0.31 | 0.49 | 0.22 |
| Decomposition | Fungi diversity | 0.34 | 0.23 | 0.13 | 0.36 | 0.31 | 0.32 |
| Decomposition | Mesoinvertebrate abundance | - | - | 0.12 | 0.41 | - | - |
| Mesoinvertebrate abundance | Temperature | - | - | 0.01 | -0.21 | - | - |
| Mesoinvertebrate abundance | Litter cover | - | - | 0.03 | -0.13 | - | - |
| Mesoinvertebrate abundance | Humidity | - | - | 0.40 | -0.06 | - | - |
| Mesoinvertebrate abundance | Treatment | - | - | 0.22 | 0.07 | - | - |


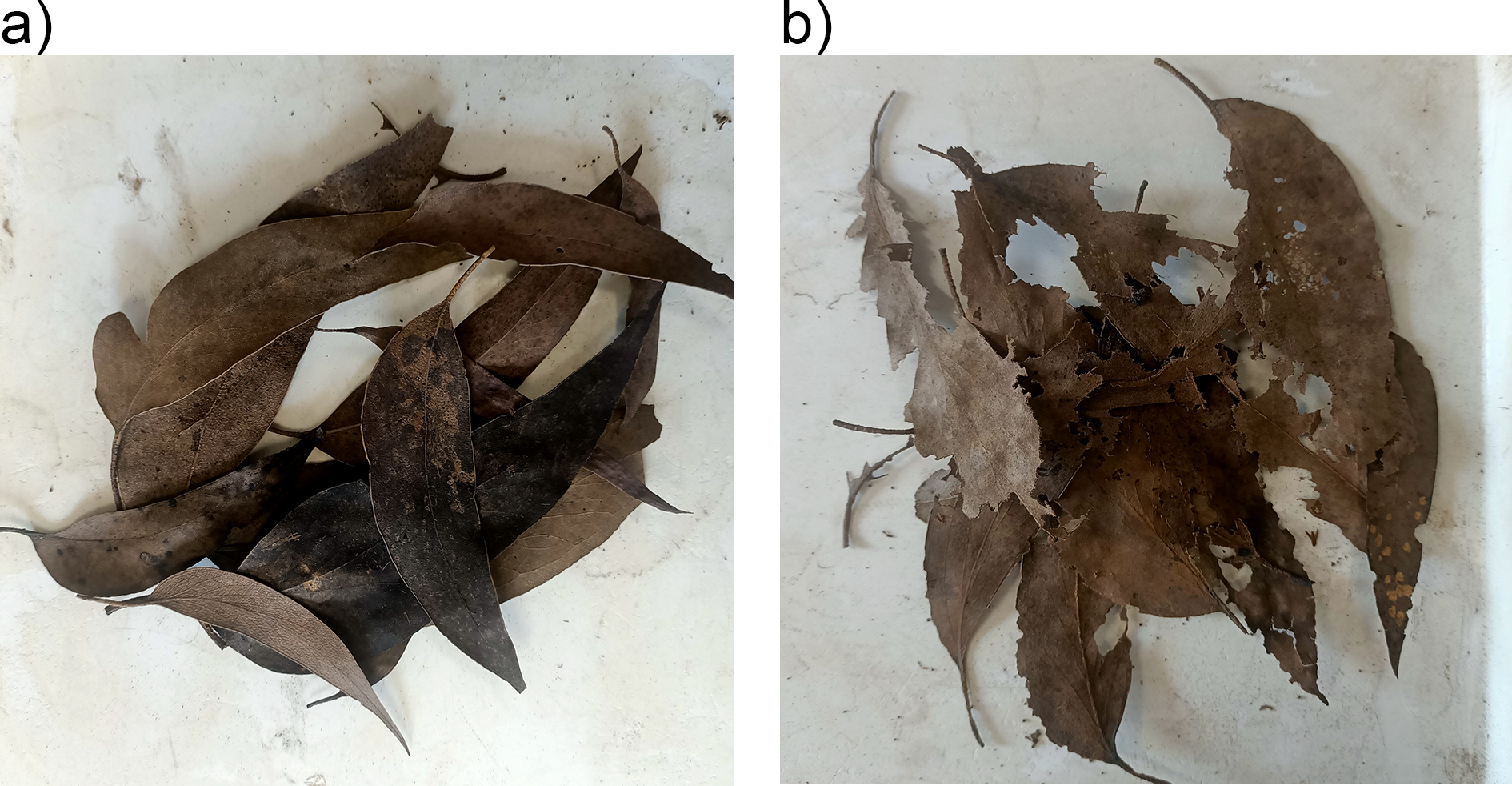
**Figure S4.** An example of undamaged leaf litter before it was left in the field (a) and evidence for leaf litter shredding by unknown macroinvertebrate detritivores after 3 months left in the field during the 35 months post-rewilding session (b).
